# Osteoarthritic Synovial Fluid Modulates Cell Phenotype and Metabolic Behavior *In Vitro*

**DOI:** 10.1155/2019/8169172

**Published:** 2019-01-15

**Authors:** Eduardo Branco de Sousa, Gilson Costa dos Santos Junior, Ramon Pinheiro Aguiar, Rafaela da Costa Sartore, Ana Carolina Leal de Oliveira, Fabio Ceneviva Lacerda Almeida, Vivaldo Moura Neto, Diego Pinheiro Aguiar

**Affiliations:** ^1^Research Division, National Institute of Traumatology and Orthopedics Jamil Haddad, Rio de Janeiro, RJ, Brazil; ^2^Program of Cell and Developmental Biology, Institute of Biomedical Sciences, Federal University of Rio de Janeiro, Rio de Janeiro, RJ, Brazil; ^3^Center of Structural Biology and Bioimaging I (CENABIO I), Federal University of Rio de Janeiro, Rio de Janeiro, Brazil; ^4^Biomedical Laboratory of the Brain, Institute of Brain Paulo Niemeyer, Rio de Janeiro, RJ, Brazil; ^5^Pharmacy Unit, West Zone State University, Rio de Janeiro, Brazil

## Abstract

Synovial fluid holds a population of mesenchymal stem cells (MSC) that could be used for clinical treatment. Our goal was to characterize the inflammatory and metabolomic profile of the synovial fluid from osteoarthritic patients and to identify its modulatory effect on synovial fluid cells. Synovial fluid was collected from non-OA and OA patients, which was centrifuged to isolate cells. Cells were cultured for 21 days, characterized with specific markers for MSC, and exposed to a specific cocktail to induce chondrogenic, osteogenic, and adipogenic differentiation. Then, we performed a MTT assay exposing SF cells from non-OA and OA patients to a medium containing non-OA and OA synovial fluid. Synovial fluid from non-OA and OA patients was submitted to ELISA to evaluate BMP-2, BMP-4, IL-6, IL-10, TNF-*α*, and TGF-*β*1 concentrations and to a metabolomic evaluation using ^1^H-NMR. Synovial fluid cells presented spindle-shaped morphology *in vitro*. Samples from OA patients formed a higher number of colonies than the ones from non-OA patients. After 21 days, the colony-forming cells from OA patients differentiated into the three mesenchymal cell lineages, under the appropriated induction protocols. Synovial fluid cells increased its metabolic activity after being exposed to the OA synovial fluid. ELISA assay showed that OA synovial fluid samples presented higher concentration of IL-10 and TGF-*β*1 than the non-OA, while the NMR showed that OA synovial fluid presents higher concentrations of glucose and glycerol. In conclusion, SFC activity is modulated by OA synovial fluid, which presents higher concentration of IL-10, TGF-*β*, glycerol, and glucose.

## 1. Introduction

Osteoarthritis (OA) involves cartilage degeneration, synovial inflammation, and subchondral bone thickening [1, 2]. Periarticular muscles, nerves, bursae, and adipose tissue are also affected, contributing to OA and to its symptoms. Recently, OA has been considered as a synovial joint disease, resulting in its failure [3, 4].

Mesenchymal stem cells (MSC) are useful in tissue regeneration and treatment of many diseases [5, 6]. MSC have already been detected in most of the tissues that compose synovial joints, such as the bone marrow, cartilage, synovial membrane, and synovial fluid [5–7]. Importantly, those cells are also detected in diseased tissues and its regenerative potential has been evaluated *in vivo* [8–12]. Knee synovium or synovial membrane presents cells that can be expanded *in vitro* and that, under appropriated conditions, can be induced to differentiate into osteogenic, chondrogenic, and adipogenic lineages. These cells are known as synovial membrane mesenchymal stem cells (SM-MSC) [13].

Synovial fluid (SF) contacts all tissues in the joint while lubricates and nourishes articular cartilage [14]. It can be easily assessed by minimally invasive aspiration [15] and holds a resident MSC population (SF-MSC) which increases in patients with OA [16]. Although SF of the osteoarthritic knee presents fewer MSC than the synovium, both have similar proliferation potential during *in vitro* expansion [17]. Besides, studies in equine [11, 18], canine [19], and humans [20] indicated that SF-MSC could be useful for treatment of cartilage defects and OA, as suggested in a review published by our group [21].

Metabolomics involves the analysis of metabolic products in biological fluids or tissue samples, which has recently been investigated as a method for OA research [22, 23] and as a promising tool for early OA diagnosis [24]. Hence, synovial fluid has been the focus of metabolomic studies in humans and animal models involving OA [25–27]. Also, it seems that metabolic profiles are related to the radiographic severity of the disease [25].

The goal of this study was to characterize the inflammatory and metabolomic profile of the synovial fluid from osteoarthritic patients and to identify its modulatory effect on synovial fluid cells.

## 2. Materials and Methods

### 2.1. Population and Collection of Synovial Fluid

The population of the study was composed of patients submitted to knee surgical treatment in the National Institute of Traumatology and Orthopedics Jamil Haddad (INTO/MS, Rio de Janeiro, Brazil). Eligible patients from both genders were divided into two groups: patients without knee OA (non-OA), Kellgren-Lawrence = 0, and patients with knee OA (OA), Kellgren-Lawrence > 2. Patients with knee infection and autoimmune diseases or submitted to prior surgery on the knee were excluded. Knee OA was graded according to Kellgren and Lawrence classification using standard standing radiographs [28]. Regarding patient safety, we could not collect synovial fluid samples from healthy individuals. So, we chose patients that underwent knee arthroscopy for treatment of anterior cruciate ligament or meniscal tear treatment as the non-OA control group. These patients had no clinical or radiographic signs of knee OA and did not fulfil any of the American College of Rheumatology criteria for OA diagnosis. The study protocol was approved by the Ethics Committee of the National Institute of Traumatology and Orthopedics (CAAE 08663912.3.0000.5273).

Synovial fluid was collected from the knee joint from non-OA patients at the beginning of the arthroscopy by syringe aspiration, just after the confection of the portals. Synovial fluid was collected from the knee joint of OA patients by syringe aspiration after arthrotomy.

### 2.2. Isolation and Culture of Synovial Fluid Cells

The synovial fluid specimens were firstly centrifuged at 16.6 *g* for five minutes at room temperature and then centrifuged at 1600 *g* for five minutes at 4°C. The supernatant was stored in 1 mL aliquots at −80°C for further analysis. The pellet from the first centrifugation was suspended in Iscove's Modified Dulbecco's Medium (IMDM, Sigma, Saint Louis, Missouri) supplemented with 10% fetal bovine serum (FBS, Gibco, Thermo Fisher). Cells were seeded at 5 to 7 × 10^5^ cells per T-75 cm^2^ culture flasks. After 24 h, the cells were washed once with serum-free medium and the medium was changed. Cell cultures were maintained at 37°C in a humidified atmosphere containing 5% CO_2_. The medium was changed every two days. Cells were harvested and replated for expansion at 80% of confluence until the 3^rd^ passage.

### 2.3. CFU Assay

2.5 × 10^5^ cells were plated and at the 14^th^ day after isolation; synovial fluid cells (SFC) from non-OA patients (*n* = 9) and OA patients (*n* = 20) were fixed with 4% paraformaldehyde solution in PBS for 30 min. Then, cells were stained with 0.1% cresyl violet for 10 min and washed with PBS for three times. Excess was removed with 70% ethanol. Photomicrographs were taken using a microscope (Nikon eclipse TS100) for the evaluation of the number and diameter of colonies. Colonies were considered when they contained more than 50 cells [29].

### 2.4. Osteogenic, Chondrogenic, and Adipogenic Differentiation In Vitro

SF cells were seeded in glass coverslips in 12-well plates at the density of 1.0 × 10^5^ cells/well and incubated for 2 h to adhere. On the other day, the medium was changed by an induction medium.

Osteogenic induction medium was composed of IMDM (Sigma-Aldrich) supplemented with 1% FBS (Gibco, Thermo Fisher), 5 *μ*g/mL L-ascorbic acid 2-phosphate sesquimagnesium salt hydrate (Sigma-Aldrich), 10 mM *β*-glycerophosphate (Sigma Saint Louis, Missouri), 1 *μ*M dexamethasone (Decadron, Roche), and 1% penicillin (Sigma-Aldrich, Saint Louis, Missouri). The medium was replaced every two days, for 21 days.

Adipogenic induction medium was composed of IMDM (Sigma-Aldrich) supplemented with 1% FBS (Gibco, Thermo Fisher), 50 mM isobutylmethylxanthine (IBMX, Sigma, Saint Louis, Missouri), 10 *μ*M insulin (Sigma, Saint Louis, Missouri), 200 *μ*M indomethacin (Sigma, Saint Louis, Missouri), 1 *μ*M dexamethasone (Decadron, Roche), and 1% penicillin (Sigma-Aldrich, Saint Louis, Missouri). The medium was replaced every two days, for 21 days.

Chondrogenic induction medium was composed of IMDM (Sigma, Saint Louis, Missouri) supplemented with 1% FBS (Gibco, Thermo Fisher), 10 ng/mL TGF-*β*1 (Peprotech), 6.25 *μ*M insulin (Sigma, Saint Louis, Missouri), 6.25 *μ*M transferrin (Sigma-Aldrich, Saint Louis, Missouri), 5 *μ*g/mL L-ascorbic acid 2-phosphate sesquimagnesium salt hydrate (Sigma-Aldrich, Saint Louis, Missouri), and 1% penicillin. The medium was replaced every two days, for 21 days.

After this period, cells were fixed in 4% paraformaldehyde solution in PBS for 1 h and then washed two times with PBS. Calcium deposits were identified by von Kossa staining. Lipid vacuoles in the cytoplasm were identified by Oil Red O staining. Extracellular matrix rich in sulphated glycosaminoglycans was visualized by alcian blue staining.

### 2.5. Flow Cytometry

Immunophenotypic characterization of SF cells at the 3^rd^ passage was performed in non-OA patients (*n* = 12) and OA patients (*n* = 12). Cells were incubated for 30 min in the dark at 4°C with the following antibodies: mouse anti-human CD105-FITC, mouse anti-human CD90-Percp-Cy5.5, mouse anti-human CD73-APC (all from BD Biosciences, San Jose, California), mouse anti-human CD146-PE (clone SHM-57, BioLegend, San Diego, EUA), mouse anti-human CD34-FITC (Dako, Glostrup, Dinamarca), and mouse anti-human CD45-Percp-Cy5.5 (clone D3/9, Immunostep, Salamanca, Espanha). The cells were acquired on an Accuri C6 flow cytometer (BD Biosciences, San Jose, CA), and the data files were analyzed using the software CSampler Accuri (BD Biosciences, San Jose, California). A minimum of 20,000 events was analyzed for each sample.

### 2.6. MTT Assay

To evaluate mitochondrial activity, about 5 × 10^3^ SF cells from non-OA patients (*n* = 2) and OA patients (*n* = 3) were seeded/well in 96-well plates. Cells were directly maintained in a medium composed of 1 : 1 (*v* : *v*) IMDM supplemented with SF from non-OA and OA patients for 24 hours. Analysis was performed by MTT assay (Sigma-Aldrich). After 24 h of incubation, the MTT solution was removed and 100 *μ*L DMSO was added. The plate was agitated for 20 min before spectrophotometric analysis at 540 nM wavelength using GloMax-Multi Detection System (Promega).

### 2.7. ELISA

Inflammatory profile of SF from non-OA and OA patients was evaluated by the concentration of IL-6, IL-10, BMP-2, BMP-4, TNF-*α*, and TGF-*β*1 using ELISA (all kits from Booster, Pleasanton, CA). Synovial fluid samples were thawed at 37°C, centrifuged for 5 min at 1600 *g* (4°C), and then diluted 1 : 1 (100 *μ*L sample : 100 *μ*L sample dilution buffer). ELISA was performed according to manufacturer's instructions. The assays were analyzed in an absorbance microplate reader Polaris (Celer Biotecnologia S.A.).

### 2.8. NMR Metabolomics

Metabolomic profile of SF from non-OA and OA patients over SF cells was evaluated using NMR-based metabolomics. SF from non-OA and OA patients was thawed and centrifuged at 4000 *g* for 20 min at room temperature. To homogenize pH and viscosity of the samples analyzed by NMR, 700 *μ*L of supernatant was mixed with 1.5 mL of 50 mM phosphate buffer pH 7.4, encompassing 10% D_2_O and 0.1 mM DSS (4,4-dimethyl-4-silapentane-1-sulfonic acid). The mixture was filtered on Amicon Ultra with 3 KD cutoff (#UFC200324), according to manufacturer's recommendations, and the flow-through was put in a NMR tube. NMR spectra from SF samples were acquired on a Bruker Avance III spectrometer operating at 400.13 MHz. ^1^H one-dimensional spectra were acquired using excitation sculpting for water suppression (ZGESGP pulse sequence), 1024 scans, TD of 65536 points in the acquisition time of 3.27 s, relaxation delay of 1.74 s, and a sweep width of 20 ppm. Two-dimensional ^1^H-^1^H-TOCSY was acquired for assignments. All spectra were processed using TopSpin 3.2 software (Bruker). CCPNMR V2 software, with a metabolomics package installed, HMDB 3.0, and BMRB were used for the assignments. We used the web interface COLMAR-TOCSY to confirm the assignments.

For multivariate analysis, ^1^H spectra were aligned, normalized by sum of intensities, 0.02 ppm binned, and scaled by the Pareto method, on AMIX software (Bruker). Multivariate statistical analyses were done on MetaboAnalyst 3.0. We used the unsupervised principal component analysis (PCA) and supervised orthogonal partial least square discriminant analysis (OPLS-DA) to create a statistical classificatory model. For univariate analysis, we did multiple *t*-test, with a *Q* value of 5%, e.g., desired false discovery rate (FDR), with the Benjamini-Krieger-Yekutieli method, assuming the same SD between control/disease samples, on GraphPad Prism 7.03 software.

## 3. Results

### 3.1. Characteristics of the Patients

We enrolled in the study 93 patients, 29 non-OA and 64 OA patients (Supplementary Table 1). Patients in the OA group had knee OA graded as moderate, Kellgren-Lawrence grade 3 (11 patients) or severe, Kellgren-Lawrence grade 4 (53 patients). All patients in the non-OA group were graded as normal, Kellgren-Lawrence grade 0. OA patients were older than non-OA patients (OA 31.93 ± 1.75 years, *n* = 29 vs. non-OA 65.21 ± 0.85 years, *n* = 64*p* < 0.0001). On the other hand, non-OA patients were taller than OA patients (non-OA 1.74 ± 0.01 m, *n* = 29 vs. OA 1.57 ± 0.01 m, *n* = 64*p* < 0.0001), which influenced on their lower BMI (non-OA 28.3 ± 1.2 kg/m^2^, *n* = 29 vs. OA 32.76 ± 0.73 m, *n* = 64*p* = 0.0024), since there was no difference between groups regarding weight (non-OA 87.73 ± 4.8 kg, *n* = 29 vs. OA 81.24 ± 1.63 m, *n* = 64*p* = 0.11).

### 3.2. Clonogenic and Morphological Characteristics

**Figure 1:**
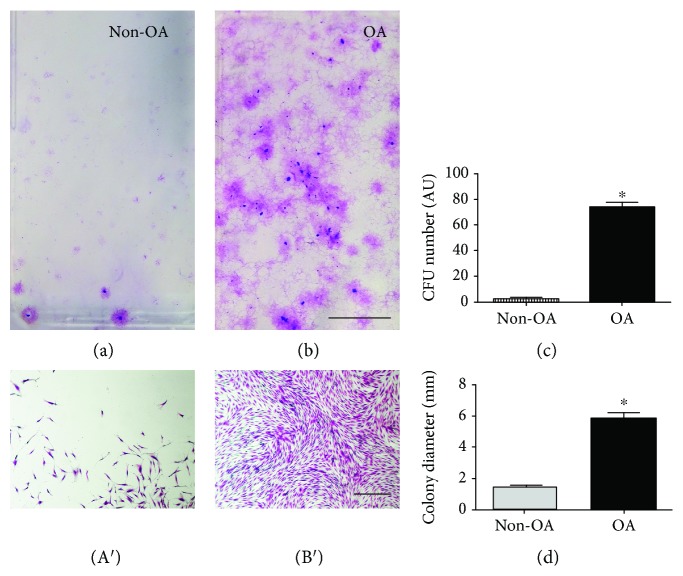
CFU phenotype. CFU stained with cresyl violet. OA patient's SFC formed more colonies (b) than non-OA ones (a). OA patient's CFU (B′) had higher diameter than non-OA CFU (A′). Scale (a, b): 1 mm. Scale (A′ and B′): 0.5 mm.

To investigate the effect of osteoarthritis on MSC population, we performed a CFU-E assay (Figures 1(a) and 1(b)). Cells isolated from the SF of patients with or without OA presented similar characteristics, exhibiting fibroblastoid spindle-shaped morphology (Figures 1 A′ and 1 B′). The samples from OA patients presented a higher number of CFU-Fs than those from the non-OA ones (OA 74.17 ± 1.07, *n* = 23 vs. non-OA 2.16 ± 0.36 CFU-Fs per flask, *p* < 0.0001, *n* = 12, Figure 1(c)). Besides, colony diameters were also significantly increased in OA patients (OA 6.62 ± 0.28 mm, *n* = 23, vs. non-OA 1.51 ± 0.17 mm, *p* < 0.0001, *n* = 12, Figure 1(d)). These results indicate that the SF from OA patients has an increased frequency of MSC; moreover, these cells present an enhanced clonogenic potential.

Following expansion in culture, SF cells from non-OA patients proliferated as adherent cells in monolayer, while SF cells from OA patients not only grew in monolayer but also grouped in clusters.

### 3.3. Evaluation of Trilineage Differentiation Potential

**Figure 2:**
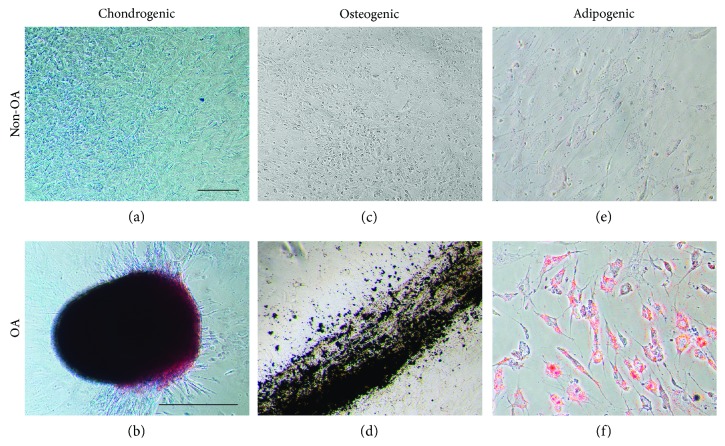
Chondrogenic, osteogenic and adipogenic induction of SFC. Images of the SFC after induction protocols. Non-OA patient's SFC did not differentiate under the appropriate protocols (a, c, e). On the other hand, OA patient's SFC differentiated, as we can identify the presence of chondroblasts evidenced by alcian blue (b), the presence of calcium deposits evidenced by von Kossa (d), and the presence of lipid accumulation with Oil Red O (f). Scale (a, c, d, e, f): 10 *μ*m. Scale (b): 500 *μ*m.

To demonstrate the multilineage potential of SF cells, chondrogenic, osteogenic, and adipogenic differentiation was induced. SF cells from non-OA patients were not able to differentiate into any induced cell types (Figures 2(a), 2(c), and 2(e)). On the other hand, SF cells from OA patients demonstrated trilineage differentiation capacity. After 21 days in differentiation media, SF cells from OA patients formed tridimensional nodules intensively stained with alcian blue (Figure 2(b)), deposited calcium visualized by von Kossa staining (Figure 2(d)), and developed into Oil Red O-positive lipid-laden fat cells (Figure 2(f)).

### 3.4. Immunophenotypic Characterization

**Figure 3:**
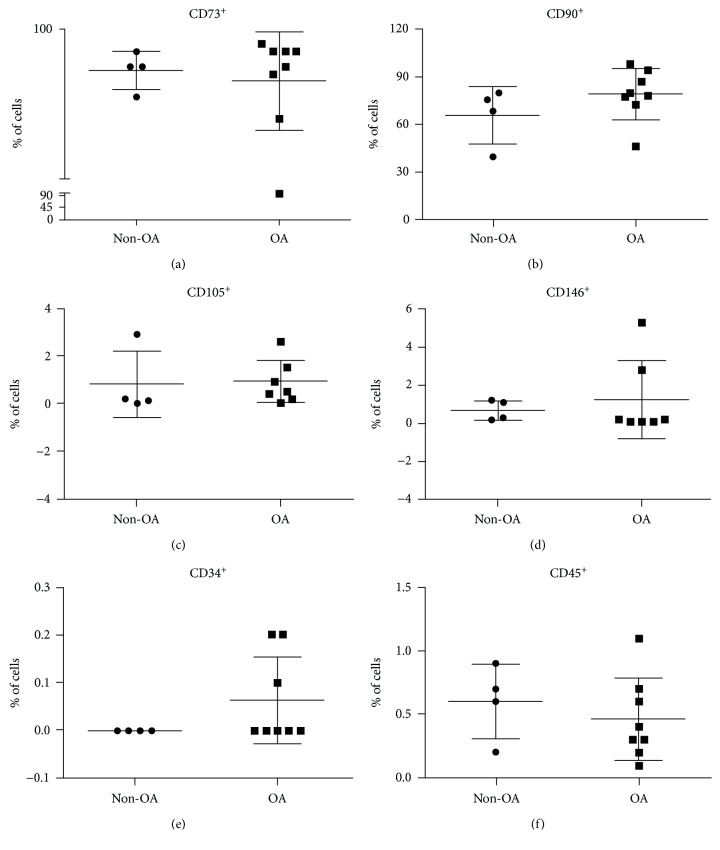
SFC flow cytometry. Isolated analysis of markers showed no differences in the concentration of SFC positive for CD73, CD90, CD146, CD34, and CD45 between non-OA and OA patients. OA SFC presented higher concentration of CD105 positive cells. Grouped analysis of markers showed no differences in the concentration of SFC positive for CD73 and CD90; CD73, CD90, and CD105; CD73, CD90, and CD146; and CD73, CD90, CD105, and CD146 between non-OA and OA patients.

For further characterization of SF cells, the immunophenotypic profile was assessed by flow cytometry. First, we evaluated the proportion of cells expressing MSC markers. There was no difference in the proportion of SF cells expressing CD45, CD73, CD90, or CD146 in OA and non-OA patients (CD34: statistical analysis not possible; CD45: 0.43 ± 0.09% vs. 0.65 ± 0.18%, respectively, *n* = 12, *p* = 0.14; CD73: 99.46 ± 0.12% vs. 99.15 ± 0.41%, respectively, *n* = 12, *p* = 0.18; CD90: 77.56 ± 5.29% vs. 69.58 ± 11.29%, respectively, *n* = 12, *p* = 0.23; CD146: 1.27 ± 0.76% vs. 0.67 ± 0.27%, respectively, *n* = 11, *p* = 0.29). In relation to CD105, there were more cells expressing this receptor in OA patients than in non-OA ones (OA 1.2 ± 0.3% vs. non-OA 0.2 ± 0.1%, *n* = 12, *p* = 0.04). No SFC from non-OA patients was positively marked for CD34, while 0.062% SFC from OA were marked (Figure 3).

Then we evaluated paired markers to assign differences between the groups. Results did not find any significant differences when comparing groups in relation to percentage of cells positive for CD73 and CD90 (non-OA 81.2 ± 6.27%, *n* = 3 vs. OA 86.44 ± 3.07%, *n* = 7, *p* = 0.42); CD73, CD90, and CD105 (non-OA 0.26 ± 0.03%, *n* = 3 vs. OA 0.21 ± 0.05%, *n* = 7, *p* = 0.54); CD73, CD90, and CD146 (non-OA 0.46 ± 0.08%, *n* = 3 vs. OA 0.64 ± 0.28%, *n* = 7, *p* = 0.35); and CD73, CD90, CD105, and CD146 (non-OA 0.15 ± 0.06%, *n* = 4 vs. OA 0.1 ± 0.03%, *n* = 7, *p* = 0.44).

### 3.5. Metabolic Activity by MTT Assay

**Figure 4:**
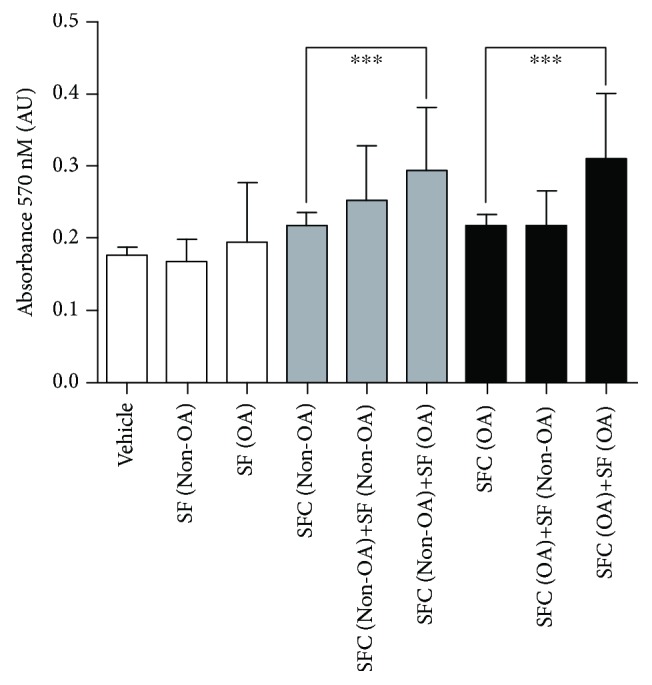
Mitochondrial activity of non-OA and OA SFC exposed to medium containing non-OA and OA SF. Medium containing non-OA and OA synovial fluid increases OA SFC (OA SFC 0.21 ± 0.002 vs. OA SFC+SF OA 0.30 ± 0.007; *p* < 0.0001, *n* = 7) and non-OA SFC activity (non-OA SFC 0.222 ± 0.003 vs. non-OA SFC+SF OA 0.30 ± 0.01; *p* = 0.0017, *n* = 7). OA: osteoarthritis.

To gain insight whether OA environment could modulate the metabolic activity of SF cells (Figure 4), we exposed SF cells isolated from non-OA patients' to OA patients' SF. As a result, we found that non-OA SF cells in contact with OA SF increased mitochondrial activity (0.222 ± 0.003 arbitrary units (AU), in control medium vs. 0.3 ± 0.01 AU in a medium supplemented with OA SF, *p* = 0.017). The same occurred for SF cells from patients with knee OA (0.21 ± 0.002 AU in a control medium vs. 0.3 ± 0.007 AU in a medium supplemented with OA SF, *p* < 0.0001). Mitochondrial activity of SF cells did not alter after exposure to SF without OA (*p* > 0.05).

### 3.6. Inflammatory Profile

**Figure 5:**
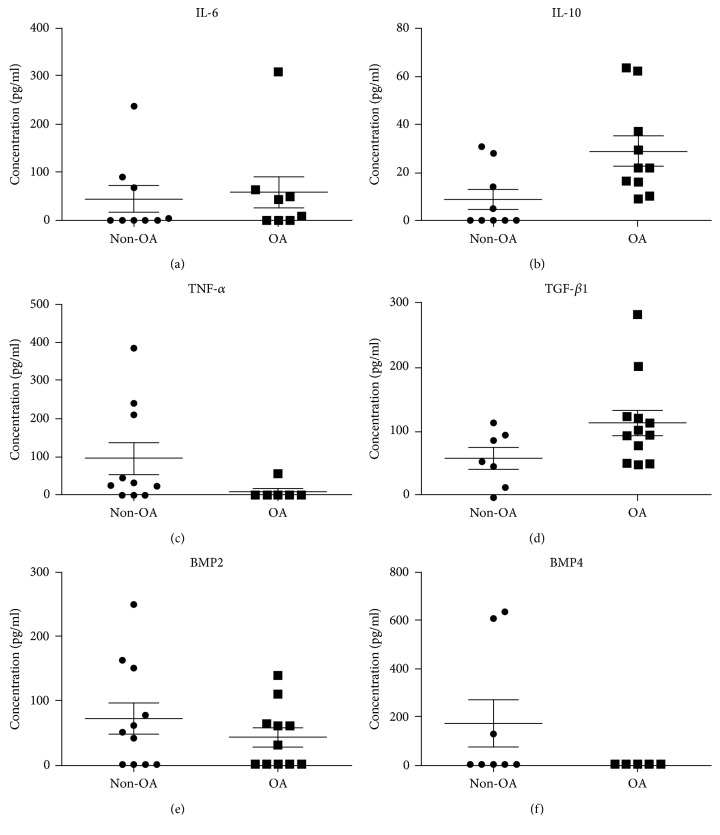
IL-6, IL-10, TNF-*α*, TGF-*β*, BMP-2, and BMP-4 concentration in non-OA and OA SF. We found no statistically significant differences between the concentrations of (a) IL-6 (non-OA SF 44.36 pg/mL ± 26.81 vs. OA SF 57.75 pg/mL ± 32.57; *p* > 0.05), (c) TNF-*α* (non-OA SF 96.76 pg/mL ± 42.44 vs. OA SF 9.55 pg/mL ± 9.55; *p* > 0.05), (e) BMP-2 (non-OA SF 72.26 pg/mL ± 24.96 vs. OA SF 42.20 pg/mL ± 14.83; *p* > 0.05), and (f) BMP-4 (below detection limits of the method) in the non-OA and OA SF. The concentrations of IL-10 (b) and TGF-*β*1 (d) were higher in the OA SF (IL-10: non-OA SF 8.65 pg/mL ± 4.23 vs. OA SF 28.93 pg/mL ± 6.32; *p* = 0.0093; TGF-*β*1: non-OA SF 58.29 pg/mL ± 16.03 vs. OA SF 113.4 pg/mL ± 19.6; *p* = 0.03). IL: interleukin; OA: osteoarthritis.

To assign whether inflammatory signaling proteins could be involved in the metabolic modulation exerted by OA SF, the amounts of IL-6, IL-10, TNF-*α*, and TGF-*β*1 were assayed (Figure 5). We found no statistically significant differences between the SF from patients with and without OA regarding the concentrations of IL-6 (OA SF 57.75 pg/mL ± 32.57 vs. non-OA SF 44.36 pg/mL ± 26.81; *n* = 18; *p* > 0.05) and TNF-*α* (OA SF 9.55 pg/mL ± 9.55 vs. non-OA SF 96.76 pg/mL ± 42.44; *n* = 16; *p* > 0.05). IL-10 and TGF-*β*1 concentrations were statistically higher in the SF from patients with OA (IL-10: OA SF 28.93 pg/mL ± 6.32 vs. non-OA SF 8.65 pg/mL ± 4.23, *n* = 19, *p* = 0.0093 and TGF-*β*1: OA SF 113.4 pg/mL ± 19. 6 vs. non-OA SF 58.29 pg/mL ± 16.03, *n* = 19, *p* = 0.03). Besides, we investigated the concentrations of BMP-2 and BMP-4. We did not detect difference in BMP-2 concentration (OA SF 42.20 pg/mL ± 14.83 vs. non-OA SF 72.26 pg/mL ± 24.96; *n* = 22; *p* > 0.05). BMP-4 concentration was inferior to the method's detection limit (*n* = 13).

### 3.7. NMR Metabolomics

NMR contributed to the understanding of the impact of the metabolic environment of the synovial fluid for the pathologies of the synovial bursae. NMR metabolomics, as performed here, gives insights on the major metabolic changes that could have a role to trigger the cellular transformation described in this manuscript. It should be clear that there is no direct assessment of the role of each metabolite.

The NMR spectra of both non-OA (*n* = 9) and OA (*n* = 31) patient's SF are very dependent on the viscosity, resulting in broad lines, which makes it very difficult for the assignment and comparison of the SF among patients. For this reason, we diluted and removed the high molecular weight component of the SF, resulting in NMR spectra with sharp lines and constant pH. We observed four major metabolites in the SF, which are in millimolar concentration: lactate, glucose, glycerol, and urea. We also observed the presence of several amino acids. We could assign and confirm in the TOCSY the presence of valine, alanine, glutamine, glutamate, lysine, phenylalanine, and tyrosine. These amino acids are in the micromolar concentration. The spectra strongly suggest (not confirmed) the presence of glycine, leucine, serine, and arginine, which are also in the micromolar range. We also detected the presence of betaine, choline, and taurine, by COLMAR-TOCSY (Supplementary Table 2 and Supplementary Figure 1) derived from the amino acid metabolism. We could not detect the presence of asparagine, aspartate, isoleucine, tryptophan, threonine, histidine, proline, methionine, and cysteine. They are either at low concentration or absent in the SF. We detected the presence of ethanol, possibly as a contaminant of the SF collection.

The main components of the SF are nutritionally important for the cellular metabolism within the synovial burse. It is interesting to note the presence of the essential amino acids phenylalanine, valine, leucine, and lysine, which cannot be biosynthesized. We also observed the presence of conditionally essential amino acids glycine, glutamine, tyrosine, and arginine, which are synthesized only in special physiopathological situations. These amino acids are unlikely to be synthesized in the synovial burse. They are most probably obtained from the blood/synovial burse barrier. Alanine, glutamate, and serine are the only nonessential amino acids that could be originated in the cellular metabolism of the cells within the synovial burse. Betaine, choline, and taurine, as well as glucose and glycerol, may act as osmolytes to protect cell membranes from stress.

**Figure 6:**
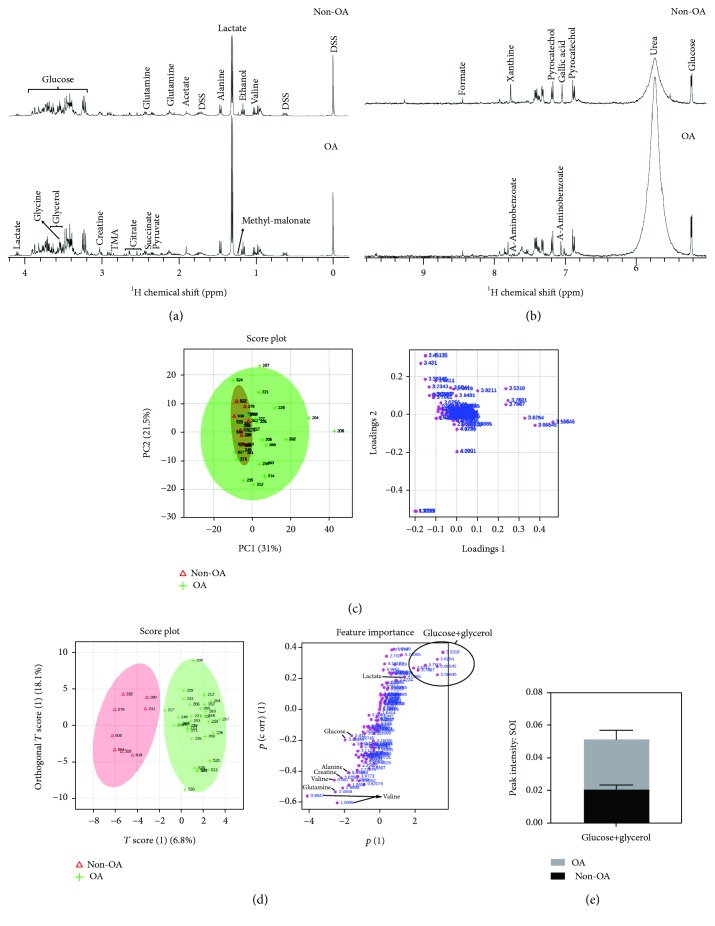
Non-OA and OA synovial fluid presents different metabolomic profiles. NMR spectra of the non-OA and OA synovial fluid in the aliphatic (a) and aromatic (b) region. The principal component analysis (PCA) score plot (c) shows the distribution of each patient due to the variance of metabolite intensity. The loading plot, on the right, shows the importance of each metabolite, i.e., the charge factor of each metabolite in class separation of non-OA (*n* = 9) and OA patients (*n* = 31). Multivariate statistical analysis, OPLS-DA (d), shows that it is possible to distinguish the group of non-OA patients from the OA ones based on the metabolomic profile. The score plot, in which each dot represents a patient, and loading plot (charge factor), on the right, in which each dot corresponds to a metabolite. Glucose and glycerol were the metabolites with most altered concentration in OA patients (e). ^∗^ *p* < 0.05.

For NMR analysis, unidimensional spectra were acquired in the hydrogen proton (^1^H), and many metabolites were evidenced at different concentrations in the SF from patients with and without OA, such as glucose, glutamine, acetate, alanine, lactate, ethanol, glycine, citrate, pyruvate, and methylmalonate (Figure 6(a), aliphatic region) and formate, xanthine, pyrocatechol, gallic acid, urea, glucose, and 4-amynobenzoate (Figure 6(b), aromatic region). Principal component analysis (PCA) was not able to classify the patients with and without OA by metabolic profile, but we could detect some subclasses of patients by the outliers shown in the PCA and univariate analysis (Supplementary Figure 2). So, we performed an orthogonal partial least square discriminant analysis (OPLS-DA), which considers the major variability between the samples, to separate them into different classes and to identify the metabolites most responsible for this (Figure 6(c)). OPLS-DA showed that glucose, glycerol, valine, and lactate were the metabolites most responsible for class separation of the patients with and without OA (Supplementary Table 3). Univariate analysis evidenced that only glycerol and glucose had statistically significant differences (*q* = 0.01) and increased more than 100% in patients with OA (Figure 6(d)). The multivariate and univariate analyses from all 124 buckets are in Supplementary Table 4.

## 4. Discussion

In this study, first we confirmed that SFC can be isolated from patients with and without knee OA and cultivated *in vitro*. In culture, these cells show plastic adherence, are spindle-shaped and form colonies, as described before [16, 30–33]. We also observed that SFC from patients with OA formed more CFU-Fs, which also presented higher diameter than the ones from patients without OA, suggesting that the first presents higher clonogenic activity. The age difference between the groups was due to the kind of surgical procedure that the patients were submitted to, since one group had knee OA and the other did not have.

Previously, it was described that intra-articular bleeding could stimulate an increase in SFC number by the recruitment of cells by cytokine and chemokine signaling [34]. However, in a long-term postlesion scenario, this increase is not sustained [30]. Given that our group without OA was composed of patients who underwent knee arthroscopy for the treatment of chronic anterior cruciate ligament (ACL) lesions and/or meniscectomy, it is possible that it did not portrait postlesion characteristic cell number. On the other hand, our OA group was composed of patients in whom the disease was in activity. SFC number is increased in patients with meniscal tear (31), ACL lesion [30], early stages of knee OA [16], temporomandibular joint (TMJ) OA [34], and osteochondral lesion of the talus [35]. In patients with meniscal tear, the number of SFC CFU-Fs is higher than that in healthy subjects [31]. However, hip SFC present less proliferative and differential potential than the ones paired from the same patient's knee [36].

ISCT defines that for a cell type to be determined as MSC, it should be plastic adherent, form CFU-Fs, and have differentiation potential to osteogenic, chondrogenic, and adipogenic lineages. Besides, it must express the cell surface markers CD40, CD44, CD73, CD90, CD105, and CD146 and must not express the hematopoietic markers CD11b, CD34, CD45, and CD271 [37]. Our results do not allow us to assert if isolated cells from the SF are typical MSC, because they do not present simultaneous positivity for all assayed MSC markers. The only marker that was strongly expressed was CD73. We also found that OA SFC were 88% positive for CD73-CD90. While most studies use cells from the 1^st^ passage and describe expression of CD73-CD90 in over 90% of SF cells [30, 38–40], we used cells from the 3^rd^ passage which could contribute to the differences observed. As MSC differentiate, they originate intermediate precursors before fully differentiation [41]. Hence, we believe that the cell type identified in this study is the same described previously, originated from a MSC, but still presenting a differentiation potential into the three mesenchymal lineages, representing a synovial progenitor cell.

Our results evidenced that SFC from patients with OA showed a higher proportion of cells expressing CD105. Nevertheless, we did not find any difference regarding other surface markers or in the association of them. Less than 2% of our SFC was positive for CD34 and CD45. Patients with TMJ dysfunction present more than 95% of SFC expressing CD44, CD73, CD90, and CD105, but negative for CD11b, CD19, CD34, CD45, CD146, HLA-DR, and STRO-1 [33, 34]. Regarding the knee joint, a study involving patients presenting ACL rupture or symptomatic OA evidenced that most SFC expressed CD44, CD73, and CD90, but not CD34 and CD45 [31]. Subtalar joint SFC at the 5^th^ passage have also been investigated in relation to the surface markers and demonstrated high expression of CD90 and CD105 besides low expression of CD14 and CD34 [32]. After induction, we verified differentiation of the SFC from patients with OA in the chondrogenic, osteogenic, and adipogenic lineages. Based on these results, we suggest that both non-OA and OA SFC have a mesenchymal origin according to the ISCT criteria [37]. Inflammatory profiling of the synovial fluid by ELISA found no differences in IL-6, TNF-*α*, BMP-2, and BMP-4 concentrations between patients with and without OA. However, IL-10 and TGF-*β*1 levels were higher in the OA synovial fluid. Although these cytokines are closely involved in OA pathophysiology, few studies addressed this profile comparison based on synovial fluid, being most of the studies performed in blood samples [42–44]. A recent study showed that IL-1*β*, IL-5, IL-6, IL-10, IL-13, and TNF-*α* concentration in plasma were significantly higher in the synovial fluid of patients with knee OA than paired samples, what could be suggested by the permeability and transport of these cytokines by the synovial membrane. Besides, the study demonstrated that IL-2, IL-4, and IL-6 concentrations were higher in OA patients compared to healthy subjects [43]. It has also been demonstrated that both circulating [42] and synovial fluid [45] IL-6 and TNF- *α* levels are highly associated with increased risk of OA in patients with history of meniscectomy. We did not find any differences in IL-6 and TNF-*α* levels. Moreover, we found that IL-10 concentration was increased in OA synovial fluid. Since IL-6 and IL-10 have opposite roles in inflammatory modulation, we believe that the raise in IL-10 level has negatively modulated the production of IL-6. Although we found no differences regarding BMP-2 concentration between the groups, it has been described that plasma and synovial fluid BMP-2 levels positively correlate with Kellgren-Lawrence classification and WOMAC score [46].

We found higher TGF-*β* levels in OA synovial fluid. TGF-*β* in the synovial fluid has an important role in MSC recruitment. Besides, OA synovial fluid stimulates OA synovial cell expansion in MSC culture by enhancing cell migration [47]. Another study found that continuous expression of TGF-*β* over synovium MSC stimulates their proliferation and chondrogenic potential [32]. TGF-*β* promotes articular cartilage growth, repair, and maintenance through regulation of its signaling pathway that targets a group of transcription and growth factors [48]. Many authors affirm that TGF-*β* has a central role in OA development [49–52]. It has also been described that normal synoviocytes protect cartilage from deleterious trauma effects and reduce the progression to an OA phenotype [53].

We demonstrated that the mitochondrial activity of the OA and non-OA SFC increases when exposed to OA, but not to non-OA synovial fluid. In one study, chondrocytes in a transwell coculture system induced a chondrogenic phenotype in synovial MSC [54]. On the other hand, OA synovial fluid delayed cartilage repair by subchondral progenitor cells in another experimental design [55]. It has also been described that normal synoviocytes protect cartilage from deleterial trauma effects and reduce the progression to an OA phenotype [53].

We believe that not only inflammatory but also synovial fluid metabolic features are potentially involved in OA SFC modulation, which needs to be further investigated. To achieve this goal, we performed a metabolomics analysis of the synovial fluid using NMR. Metabolomics involves the study of the metabolites present in a biological system [15], allowing to evaluate the organism response to environmental stimuli [56], and may be useful as a tool to find an early OA biomarker [24].

Using NMR, we found that the major metabolites (lactate, glucose, glycerol, and urea) have nutritional and protective roles, as osmolytes. We also observed the presence of amino acids that were imported from the blood to the synovial liquid. Glycerol levels increased in the SF of OA patients. This increase may be related to protection against the stress of the inflammatory process or have a specific metabolic consequence. One suggestion would be an increase in lipid metabolism, which has an important role as an energy source in a joint with OA due to increased lipoprotein metabolism secondary to hypoxia [57, 58]. Two other studies have found elevation in the glycerol levels in canine [57] and equine [58] OA models. Recently, a study in humans verified that high synovial fluid glycerol levels were related to late knee OA stages corresponding to Kellgren-Lawrence stages 3 and 4 [25]. A lipidomic study verified also that glycerophospholipids were raised 3.5 times in the synovial fluid from patients with severe OA [59]. These findings are like ours, since our OA group was composed of patients whose OA was classified as Kellgren-Lawrence stages 3 and 4 and who underwent total knee replacement.

Synovial fluid metabolic profile alters according to OA severity, and one hypothesis includes mTOR inhibition and autophagy activation, involving changes in glycerolipidic metabolism, including glycerol, glycerol-3-phosphate, and fatty acid synthesis, as protective mechanisms for maintaining OA articular cartilage [25]. Another possible explanation to the significant increase in metabolites found in our study is a modification of SFC metabolism due to the high concentration of reactive oxygen species secondary to glucose oxidation by the articular cartilage OA chondrocytes and the highly proliferative SFC. We were able to demonstrate OA and non-OA SFC proliferation in cell culture and confirmed this by the increase in SFC activity when exposed to OA synovial fluid. The Warburg effect, in which a population of cells shifts its metabolism from the oxidative pathway to glycolysis, has already been described for proliferative cells, mainly hematopoietic stem cells but also bone marrow MSC, and may help explain these findings [60, 61]. Oxidative phosphorylation induces a raise in MSC senescence, while its proliferation is increased in normal oxygen conditions. Hence, hypoxia and glycolysis are necessary to avoid senescence and proliferation induced by reactive oxygen species, to maintain the long-term proliferative ability of bone marrow MSC [61].

In early stages of the disease, the metabolites found seem to be related to glycolysis, preceding the shift seen in the OA late stages to preserve glucose, indicating that metabolism in the late stages converges to energy maintenance [25]. The presence of stem cells in adult tissues may contribute to abnormal regeneration, because they are activated, but not under adequate differentiation regulation [9]. Besides, inflammatory cytokines are potentially involved in the suppression of chondrogenic ability of MSC exposed to inflammation [41]. This SFC differentiation impairment was previously observed, and it was suggested that it was caused by a problem in cell to cell communication [62].

**Figure 7:**
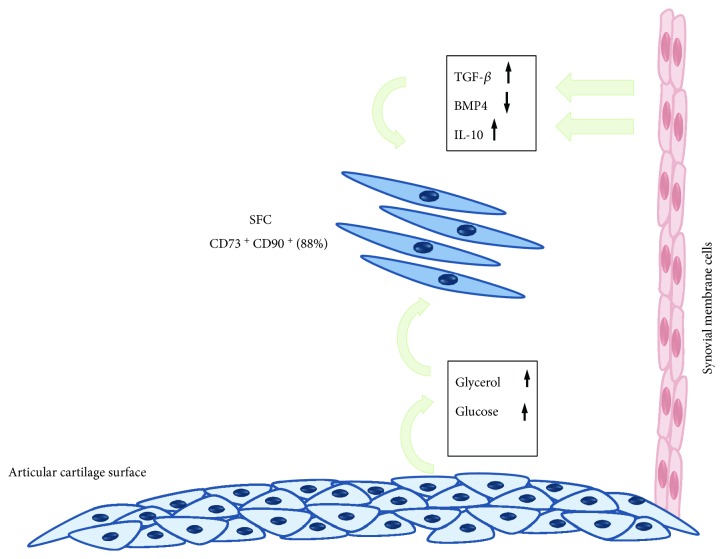
Crosstalk between articular cartilage, synovium, and OA synovial fluid cells. Articular chondrocytes altered metabolism, shifting from the anaerobic to the glycolytic pathway, and other alternative sources of energy production, leading to a raise in glycerol and glucose concentration in the synovial fluid OA SF. Besides, synovium inflammation increases TGF-*β* and IL-4 production and reduces BMP-4 expression. These alterations are interpreted by the SFC as stimuli to keep proliferation and not to differentiate. So, synovial fluid cells keep proliferating, instead of repairing articular lesions.

Our results lead us to suggest that inflammatory and metabolic alterations would be occurring in parallel, maintaining SFC in a high proliferative state but low differentiation potential in its lineage, limiting its ability to reverse the OA process (Figure 7).

## 5. Conclusion

SFC activity is modulated by OA synovial fluid, which presents higher concentration of IL-10, TGF-*β*, glycerol, and glucose.

## Data Availability

The data used to support the findings of this study are restricted by the CEP/INTO (Ethics Institutional Board) in order to protect patient's privacy. Metabolomics data are available in Supplementary Materials.

## Supplementary Materials

Supplementary Materials Table S1: patient demographics. Ninety-three patients were included in the study, 29 without knee OA and 64 with knee OA. W/O: without; W/: with; BMI: body mass index; OA: osteoarthritis; N/A: statistics not applicable. Figure S1: the ^1^H-^1^H TOCSY could confirm the presence of betaine and cadaverine. By COLMAR-TOCSY query (http://spin.ccic.ohio-state.edu/index.php/tocsy), we could assign betaine (above) and cadaverine (below) in the SF. The red circles represent the database chemical shift, the pink circles represent the experimental chemical shift, and each dark dot represents one peak. Figure S2: the metabolic profile reveals subclasses of patients. The graph on the top shows the normalized peak intensities (dots) for all buckets, and for all patients with (disease: color red), and without OA (control: black color). Some metabolites show much more variance, such as lactate, acetamide, glucose, and glycerol, due to outlier patients. On the top right, principal component analysis (PCA) was not able to classify the patients with (disease) and without OA (control) by the metabolic profile, but we could detect some subclasses of patients by the outlier patients shown in the PCA, such as patients 504, 208, 257, 516, 235, 204, and 202. The graphs below shows the outlier patients by each metabolite, e.g., valine, lactate, acetamide, and glucose+glycerol. All metabolites have the chemical shift value on the side. Metabolites. The PCA score plot shows the distribution of each patient due to the variance of metabolite intensity. The loading plot, below, shows the importance of each metabolite, i.e., the charge factor of each metabolite in class separation from patients without OA (*n* = 9) and with OA (*n* = 31). Table S2: peak report table generated by COLMAR-TOCSY query. From the COLMAR-TOCSY query, same as Supplementary Figure 2, we exported this table. The first two columns show the proton chemical shift peak values, the third shows the peak amplitude, and last two show the matched compound names with the database. In case of overlapping compounds, the table will show more than one compound name. Some peak regions without any assignment were erased. Table S3: metabolites assigned by NMR metabolomics. The table shows the assigned metabolites, the proton chemical shift values in ppm, the *q*-value from multiple *t*-test with 5% FDR approach, the difference (fold change between disease and control), the loading (t1), and the ortholoading (to1), from OPLS-DA. Table S4: result of univariate and multivariate analysis for each bucket. From the left to right column, metabolite name or if it is unknown, the ^1^H chemical shift value in ppm is written. *P* value, difference, *t* ratio, df, and *q* value were generated by multiple *t*-test, with 5% FRD approach, assuming the same SD between classes and peak intensities. Loading (t1) and ortholoading (to1) were generated by OPLS-DA.
