# Supplementary material for: Osteoarthritic Synovial Fluid Modulates Cell Phenotype and Metabolic Behavior In Vitro
Source: Stem Cells Int. 2019 Jan 15;2019:8169172. doi: 10.1155/2019/8169172 (PMC6350599; doi:10.1155/2019/8169172)
Supplement: Supplementary Materials — Table S1: patient demographics. Ninety-three patients were included in the study, 29 without knee OA and 64 with knee OA. W/O: without; W/: with; BMI: body mass index; OA: osteoarthritis; N/A: statistics not applicable. Figure S1: the 1H-1H TOCSY could confirm the presence of betaine and cadaverine. By COLMAR-TOCSY query (http://spin.ccic.ohio-state.edu/index.php/tocsy), we could assign betaine (above) and cadaverine (below) in the SF. The red circles represent the database chemical shift, the pink circles represent the experimental chemical shift, and each dark dot represents one peak. Figure S2: the metabolic profile reveals subclasses of patients. The graph on the top shows the normalized peak intensities (dots) for all buckets, and for all patients with (disease: color red), and without OA (control: black color). Some metabolites show much more variance, such as lactate, acetamide, glucose, and glycerol, due to outlier patients. On the top right, principal component analysis (PCA) was not able to classify the patients with (disease) and without OA (control) by the metabolic profile, but we could detect some subclasses of patients by the outlier patients shown in the PCA, such as patients 504, 208, 257, 516, 235, 204, and 202. The graphs below shows the outlier patients by each metabolite, e.g., valine, lactate, acetamide, and glucose+glycerol. All metabolites have the chemical shift value on the side. Metabolites. The PCA score plot shows the distribution of each patient due to the variance of metabolite intensity. The loading plot, below, shows the importance of each metabolite, i.e., the charge factor of each metabolite in class separation from patients without OA (n = 9) and with OA (n = 31). Table S2: peak report table generated by COLMAR-TOCSY query. From the COLMAR-TOCSY query, same as Supplementary Figure 2, we exported this table. The first two columns show the proton chemical shift peak values, the third shows the peak amplitude, and last two show the m [file 8169172.f1.docx]

**Supplementary table 1. Patients demographics.** Ninety-three patients were included in the study, 29 without knee OA and 64 with knee OA. W/O: without; W/: with; BMI: body-mass index; OA: osteoarthritis; N/A: statistics not applicable.

|  | W/O OA (n=29) | W/ OA (n=64) | *p* value |
| --- | --- | --- | --- |
| GENDER | Male: 24  Female: 5 | Male: 12  Female: 52 | N/A |
| WEIGHT (Kg) | 87.73 ± 4.8 | 81.24 ± 1.63 | *p*=0.11 |
| HEIGHT (m) | 1.74 ± 0.01 | 1.57 ± 0.01 | *p*<0.0001 |
| BMI (Kg/m^2^) | 28.3 ± 1.2 | 32.76 ± 0.73 | *p*=0.0024 |
| AGE (years) | 31.93 ± 1.75 | 65.21 ± 0.85 | *p*<0.0001 |
| LATERALITY | Right: 15  Left: 14 | Right: 35  Left: 29 | N/A |
| OA GRADE | Grade 0: 29 | Grade 3: 11  Grade 4: 53 | N/A |

**Supplementary figure 1. The ^1^H-^1^H TOCSY could confirm the presence of betaine and cadaverine.** By COLMAR-TOCSY query (<http://spin.ccic.ohio-state.edu/index.php/tocsy>), we could assign betaine (above) and cadaverine (below) in the SF. The red circles represent the database chemical shift, the pink circles represent the experimental chemical shift, and each dark dot represent one peak.


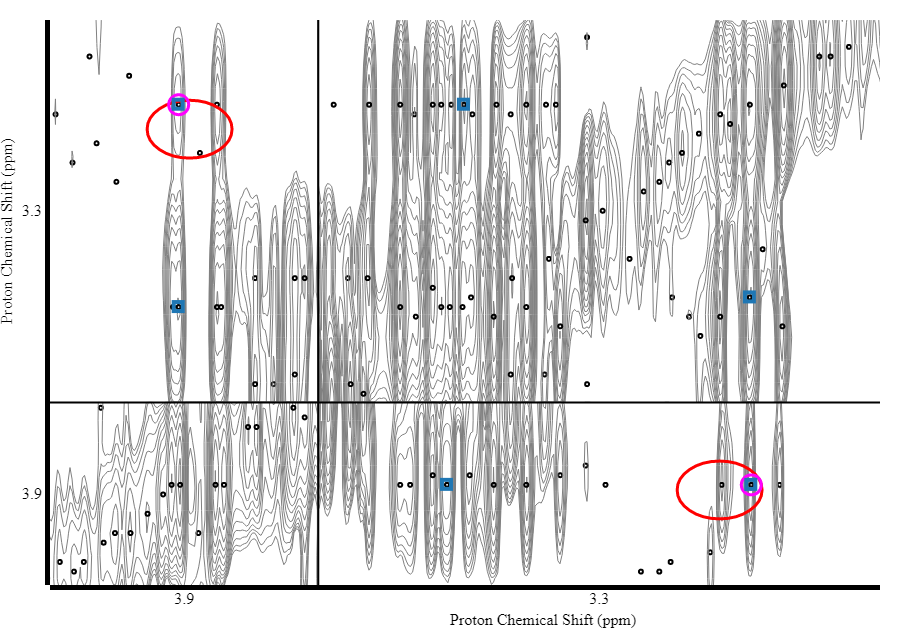

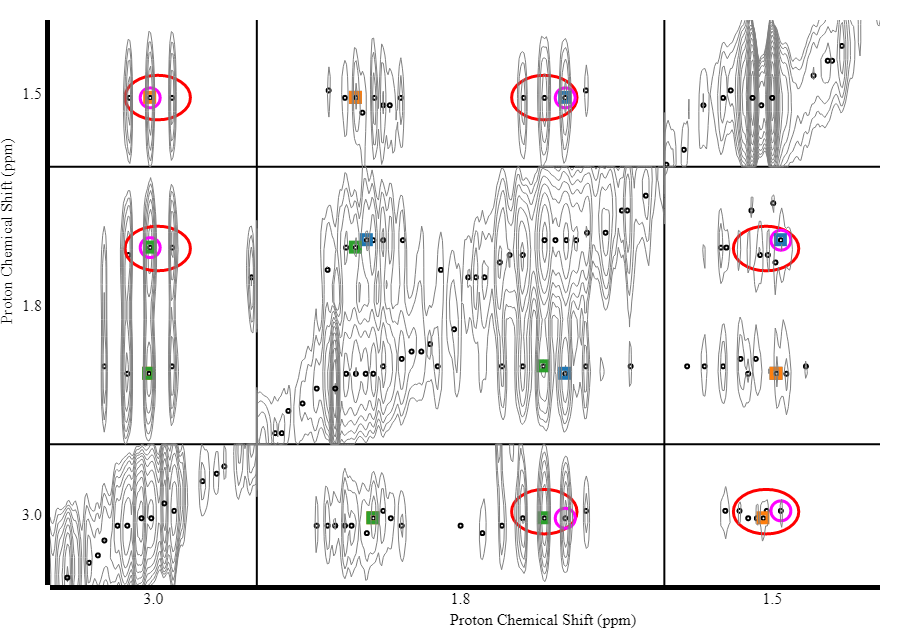


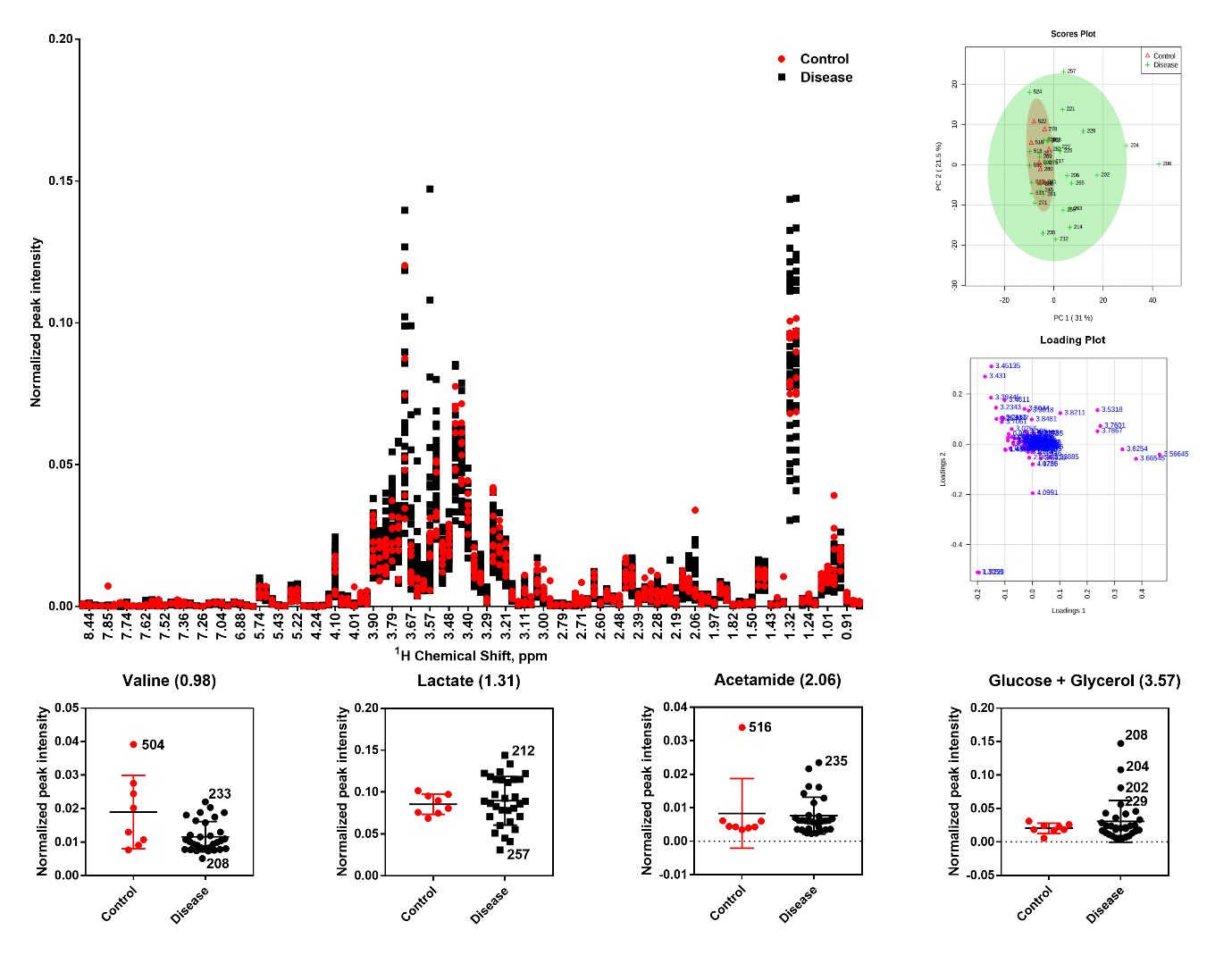


**Supplementary Figure 2. The metabolic profile reveals subclasses of patients.** The graph on the top, shows the normalized peak intensities (dots) for all buckets, and for all patients with (Disease-color red), and without OA (Control-Black color). Some metabolites show much more variance, as lactate, acetamide, glucose and glycerol, due to outlier patients. On the top right, Principal Component Analysis (PCA) was not able to classify the patients with (Disease) and without OA (Control) by the metabolic profile, but we could detect some subclasses of patients by the outliers patients shown in the PCA, as patients 504, 208, 257, 516, 235, 204 and 202. The graphs below, shows the outliers patients by each metabolite, e.g. valine, lactate, acetamide and glucose + glycerol. All metabolites have the chemical shift value on the side. Metabolites. The PCA score-plot shows the distribution of each patient due to the variance of metabolites intensity. The Loading-plot, below, shows the importance of each metabolite, i.e., the charge factor of each metabolite in class separation from patients without OA (n=9) and with OA (n=31).

**Supplementary table 2. Peak report table generated by COLMAR-TOCSY query.** From the COLMAR-TOCSY query, same as **supplementary figure 2**, we exported this table. The first two columns show the proton chemical shift peak values, the third shows the peak amplitude, and last two, the matched compound names with the database. In case of overlap compounds, the table will show more than one compound name. Some peak regions without any assignment were erased.

| **Proton** | **Proton** | **Amplitude** | **compound_name 1** | **compound_name 2** |
| --- | --- | --- | --- | --- |
| -0.008 | -0.02 | 41332.91 |  |  |
| -0.002 | 3.62 | 10986.47 |  |  |
| -0.001 | -0.19 | 16887.77 |  |  |
| 0 | 0 | 1525498.57 |  |  |
| 0.001 | 9.39 | 11313.35 |  |  |
| 0.001 | 0.18 | 20445.48 |  |  |
| 0.002 | 7.73 | 12536.51 |  |  |
| 0.002 | 4.69 | 14256.86 |  |  |
| 0.002 | 7.31 | 14127.02 |  |  |
| 0.053 | 0.05 | 23067.87 |  |  |
| 0.077 | 0.07 | 19306.2 |  |  |
| 0.148 | 0.15 | 22704.69 |  |  |
| 0.603 | 2.9 | 36580.55 |  |  |
| 0.604 | 1.75 | 33783.9 |  |  |
| 0.605 | 0.62 | 23305.89 |  |  |
| 0.619 | 0.62 | 18222 |  |  |
| 0.619 | 0.62 | 18120.28 |  |  |
| 0.625 | 0.63 | 17532.1 | DSS_1 |  |
| 0.625 | 2.9 | 33115.95 | clique_0004 | DSS_1 |
| 0.626 | 1.75 | 24321.24 | clique_0004 |  |
| 0.632 | 0.62 | 13842.63 |  |  |
| 0.647 | 0.63 | 26908.85 |  |  |
| 0.648 | 1.75 | 35587.8 |  |  |
| 0.648 | 2.9 | 38700.6 |  |  |
| 0.812 | 0.82 | 12867.54 |  |  |
| 0.812 | 0.94 | 11401.39 |  |  |
| 0.829 | 0.83 | 14312.84 |  |  |
| 0.83 | 0.93 | 11605.66 |  |  |
| 0.842 | 0.85 | 11659.93 |  |  |
| 0.851 | 0.87 | 16338.17 |  |  |
| 0.866 | 1.69 | 32778.68 |  |  |
| 0.866 | 3.98 | 22513.35 |  |  |
| 0.867 | 0.88 | 88040.11 |  |  |
| 0.885 | 3.98 | 19966.47 |  |  |
| 0.885 | 1.68 | 32197.97 |  |  |
| 0.886 | 0.89 | 243684.18 |  |  |
| 0.904 | 1.45 | 21520.9 |  |  |
| 0.905 | 0.91 | 152300.62 |  |  |
| 0.905 | 3.98 | 18024.98 |  |  |
| 0.905 | 1.69 | 30456.13 |  |  |
| 0.916 | 0.92 | 93411.38 |  |  |
| 0.916 | 2.59 | 13858.72 |  |  |
| 0.923 | 1.95 | 13295.39 |  |  |
| 0.923 | 1.24 | 21485.33 |  |  |
| 0.923 | 1.46 | 32722.01 |  |  |
| 0.923 | 0.93 | 285774.98 |  |  |
| 0.933 | 1.69 | 74347.03 |  |  |
| 0.933 | 0.94 | 402591.61 |  |  |
| 0.934 | 3.72 | 21846.98 |  |  |
| 0.943 | 1.46 | 24720.06 |  |  |
| 0.943 | 1.24 | 19596.9 |  |  |
| 0.945 | 1.69 | 112146.21 |  |  |
| 0.945 | 1.89 | 20609.05 |  |  |
| 0.945 | 0.94 | 626256.21 |  |  |
| 0.946 | 3.7 | 48961.07 |  |  |
| 0.956 | 1.35 | 12018.1 | 2_Aminobutyric_acid_1 |  |
| 0.958 | 3.71 | 17468.23 | small_0008 |  |
| 0.96 | 1.69 | 65023.05 |  |  |
| 0.963 | 2.25 | 63450.15 |  |  |
| 0.964 | 0.99 | 962815.5 |  |  |
| 0.965 | 3.59 | 49007.96 |  |  |
| 0.966 | 1.88 | 17872.17 | 4_Aminoantipyrine_1 |  |
| 0.973 | 0.96 | 61369.99 |  |  |
| 0.973 | 1.35 | 12876.42 |  |  |
| 0.983 | 3.59 | 49083.39 |  |  |
| 0.983 | 0.99 | 1041155.58 |  |  |
| 0.984 | 2.25 | 71805.53 |  |  |
| 0.984 | 1.94 | 34657.22 |  |  |
| 0.985 | 1.25 | 17959.65 |  |  |
| 0.985 | 1.45 | 15686.93 |  |  |
| 1.002 | 1.45 | 16756.35 |  |  |
| 1.002 | 1.25 | 19740.75 |  |  |
| 1.002 | 3.65 | 15428.73 |  |  |
| 1.003 | 0.99 | 255511.06 |  |  |
| 1.003 | 1.96 | 27849.78 | L_Valine_1 | 2_Ethylacrylic_acid_1 |
| 1.015 | 2.25 | 67788.29 | clique_0003 |  |
| 1.016 | 1 | 823337.32 | L_Valine_1 |  |
| 1.016 | 3.58 | 48337.1 | clique_0003 |  |
| 1.034 | 3.58 | 45463 |  |  |
| 1.034 | 1 | 839719.82 |  |  |
| 1.035 | 2.25 | 72255.44 |  |  |
| 1.049 | 1.04 | 42643.66 |  |  |
| 1.39 | 1.39 | 56521.62 |  |  |
| 1.399 | 1.41 | 30698.01 |  |  |
| 1.403 | 1.41 | 35801.52 |  |  |
| 1.416 | 1.43 | 31814.58 |  |  |
| 1.422 | 1.25 | 12684.11 |  |  |
| 1.423 | 1.87 | 11809.83 |  |  |
| 1.426 | 1.25 | 12326.49 |  |  |
| 1.438 | 1.25 | 11896.29 |  |  |
| 1.439 | 0.96 | 10947.97 |  |  |
| 1.441 | 1.88 | 16914.63 | L_Glutamic_acid_1 |  |
| 1.445 | 1.25 | 12958.56 |  |  |
| 1.446 | 3.01 | 12309.78 | Cadaverine_1 | Lysine_1 |
| 1.446 | 1.7 | 16010.23 | clique_0012 | alpha,epsilon_  Diaminopimelic_acid_1 |
| 1.45 | 1.88 | 17051.01 | clique_0012 | clique_0007 |
| 1.451 | 1.73 | 14581.98 |  |  |
| 1.453 | 3.76 | 158281.44 | L_Glutamic_acid_1 |  |
| 1.453 | 1.65 | 14132.44 |  |  |
| 1.454 | 1.46 | 1156223.48 |  |  |
| 1.455 | 1.28 | 21934.38 |  |  |
| 1.458 | 1.72 | 13740.61 |  |  |
| 1.459 | 3.01 | 12309.83 | L_Glutamic_acid_1 |  |
| 1.459 | 0.95 | 13106.43 |  |  |
| 1.462 | 3.02 | 13117.28 | clique_0007 |  |
| 1.464 | 1.47 | 85977.82 |  |  |
| 1.465 | 1.72 | 16067.83 |  |  |
| 1.468 | 3.02 | 11831.33 |  |  |
| 1.469 | 1.86 | 15327.85 |  |  |
| 1.47 | 2.16 | 10494.28 |  |  |
| 1.472 | 1.46 | 1243551.94 |  |  |
| 1.473 | 3.76 | 146761.37 |  |  |
| 1.473 | 1.66 | 11931.13 |  |  |
| 1.474 | 1.29 | 23122.37 |  |  |
| 1.476 | 3.02 | 11342.32 |  |  |
| 1.476 | 1.88 | 16928.42 |  |  |
| 1.483 | 1.86 | 20276.85 |  |  |
| 1.484 | 3.01 | 11793.38 |  |  |
| 1.492 | 1.45 | 25804.85 |  |  |
| 1.496 | 1.71 | 13114.38 |  |  |
| 1.497 | 3.01 | 13774.5 |  |  |
| 1.499 | 1.87 | 20242.89 |  |  |
| 1.499 | 1.46 | 28205.45 |  |  |
| 1.501 | 1.71 | 19195.04 |  |  |
| 1.516 | 1.87 | 14711.21 |  |  |
| 1.517 | 1.47 | 16048.2 |  |  |
| 1.532 | 1.87 | 10931.34 |  |  |
| 1.535 | 1.53 | 13522.99 |  |  |
| 1.551 | 1.55 | 19029.42 |  |  |
| 1.561 | 1.56 | 39567.51 |  |  |
| 1.572 | 1.57 | 11210.83 |  |  |
| 1.582 | 1.58 | 14062.07 |  |  |
| 1.6 | 1.62 | 15730.17 |  |  |
| 1.618 | 1.64 | 26465.6 |  |  |
| 1.632 | 1.87 | 12685.19 |  |  |
| 1.635 | 0.93 | 22566.9 |  |  |
| 1.635 | 1.66 | 42945.79 |  |  |
| 1.64 | 1.66 | 21403.73 |  |  |
| 1.655 | 0.94 | 32663.29 |  |  |
| 1.655 | 1.69 | 50188.85 |  |  |
| 1.658 | 3.22 | 12590.03 |  |  |
| 1.659 | 3.73 | 12546.42 |  |  |
| 1.67 | 0.93 | 26683.74 |  |  |
| 1.672 | 1.69 | 62107.29 |  |  |
| 1.673 | 1.45 | 14344.47 |  |  |
| 1.673 | 3.01 | 18406.17 |  |  |
| 1.673 | 1.87 | 27349.59 |  |  |
| 1.674 | 3.22 | 13523.6 |  |  |
| 1.678 | 0.94 | 25777.36 |  |  |
| 1.681 | 3.72 | 17061.7 |  |  |
| 1.682 | 1.7 | 64145.14 |  |  |
| 1.683 | 0.94 | 35900.03 |  |  |
| 1.689 | 3.71 | 18984.02 |  |  |
| 1.691 | 1.7 | 96542.38 |  |  |
| 1.691 | 3.22 | 10977.3 |  |  |
| 1.692 | 3.02 | 47355.72 | Cadaverine_1 | Lysine_1 |
| 1.692 | 1.46 | 33583.6 | clique_0012 | Cyclohexanone_1 |
| 1.692 | 1.88 | 44666.28 | clique_0012 |  |
| 1.7 | 0.94 | 47043.7 |  |  |
| 1.701 | 1.7 | 69370.25 |  |  |
| 1.711 | 3.71 | 29355.53 | clique_0010 | L_Glutamic_acid_1 |
| 1.711 | 0.94 | 45756.34 |  |  |
| 1.711 | 1.7 | 137328.82 |  |  |
| 1.711 | 1.46 | 37976.81 | N_alpha__Acetyl_ornithine_1 | N_alpha__Acetyl_ornithine_1 |
| 1.711 | 3.02 | 61181.51 | clique_0005 | N_alpha__Acetyl_ornithine_1 |
| 1.712 | 1.87 | 45259.38 | clique_0005 | clique_0010 |
| 1.718 | 0.94 | 31121.23 |  |  |
| 1.73 | 1.46 | 26411.64 |  |  |
| 1.731 | 3.02 | 44748.66 |  |  |
| 1.731 | 1.87 | 37224.81 |  |  |
| 1.731 | 3.72 | 16649.5 |  |  |
| 1.731 | 1.72 | 91623.14 |  |  |
| 1.735 | 0.94 | 38605.25 |  |  |
| 1.741 | 2.9 | 14965.13 |  |  |
| 1.743 | 1.72 | 31499.82 |  |  |
| 1.749 | 0.93 | 22632.01 |  |  |
| 1.75 | 3.72 | 14336.55 |  |  |
| 1.75 | 1.87 | 24887.79 |  |  |
| 1.75 | 3.03 | 21207.31 |  |  |
| 1.752 | 1.73 | 46175.72 | DSS_1 |  |
| 1.752 | 0.62 | 13294.56 | clique_0004 | DSS_1 |
| 1.753 | 2.9 | 28765.57 | clique_0004 |  |
| 1.765 | 2.91 | 10943.31 |  |  |
| 1.766 | 1.75 | 24215.1 |  |  |
| 1.768 | 3.04 | 14079.18 |  |  |
| 1.774 | 1.75 | 28355.49 |  |  |
| 1.775 | 2.91 | 10504.89 |  |  |
| 1.781 | 1.75 | 16047.17 |  |  |
| 1.788 | 3.03 | 10934.08 |  |  |
| 1.794 | 1.82 | 18595.61 |  |  |
| 1.796 | 0.63 | 13015.32 |  |  |
| 1.806 | 1.74 | 14824.03 |  |  |
| 1.809 | 1.87 | 18273.72 |  |  |
| 1.816 | 1.84 | 16892.31 |  |  |
| 1.824 | 1.85 | 20958.31 |  |  |
| 1.833 | 1.85 | 24765.97 |  |  |
| 1.841 | 3.72 | 13006.06 |  |  |
| 1.841 | 1.7 | 22091.06 |  |  |
| 1.842 | 3.03 | 16747.03 |  |  |
| 1.842 | 1.86 | 40048.01 |  |  |
| 1.843 | 1.46 | 18305.36 |  |  |
| 1.851 | 3.72 | 14210.54 |  |  |
| 1.852 | 3.21 | 11900.6 |  |  |
| 1.852 | 3.02 | 14590.6 |  |  |
| 1.853 | 1.47 | 15830.19 |  |  |
| 1.859 | 1.87 | 72658.8 |  |  |
| 1.859 | 3.71 | 21310.2 |  |  |
| 1.859 | 1.47 | 22847.21 |  |  |
| 1.859 | 1.7 | 33109.37 |  |  |
| 1.859 | 3.01 | 19885.42 |  |  |
| 1.867 | 1.46 | 26081.08 |  |  |
| 1.867 | 3.71 | 31005.51 |  |  |
| 1.868 | 1.88 | 79567.36 | N_alpha__Acetyl_ornithine_1 |  |
| 1.868 | 3.02 | 26556.07 | clique_0005 | clique_0006 |
| 1.868 | 1.7 | 39960.11 |  |  |
| 1.869 | 3.23 | 15231.58 |  |  |
| 1.874 | 3.22 | 15386.66 | clique_0009 |  |
| 1.874 | 3.04 | 20120.31 | Cyclohexanone_1 | Lysine_1 |
| 1.874 | 1.7 | 31833.61 | clique_0012 |  |
| 1.875 | 3.71 | 23492.44 | L_Glutamic_acid_1 |  |
| 1.875 | 1.88 | 74125.97 |  |  |
| 1.878 | 1.48 | 15677.95 | alpha,epsilon_  Diaminopimelic_acid_1 | Lysine_1 |
| 1.884 | 1.46 | 23531.01 | clique_0012 | clique_0007 |
| 1.884 | 1.88 | 86344.52 |  |  |
| 1.884 | 1.71 | 32541.72 | clique_0005 | clique_0010 |
| 1.884 | 3.71 | 28586.7 | clique_0009 | clique_0010 |
| 1.888 | 3.03 | 24812.81 |  |  |
| 1.892 | 3.23 | 13127.89 |  |  |
| 1.893 | 1.71 | 32637.66 | alpha,epsilon_  Diaminopimelic_acid_2 | Tetrahydrofuran_1 |
| 1.893 | 3.72 | 26298.5 | clique_0006 |  |
| 1.893 | 1.88 | 70500.82 |  |  |
| 1.894 | 1.46 | 15473.75 |  |  |
| 1.894 | 3.03 | 32727.51 |  |  |
| 1.9 | 3.22 | 12295.03 |  |  |
| 1.9 | 3.71 | 14026.33 |  |  |
| 1.903 | 1.9 | 479261.51 |  |  |
| 1.903 | 3.03 | 16441.08 |  |  |
| 1.909 | 1.45 | 13163.67 |  |  |
| 1.91 | 1.74 | 22040.35 |  |  |
| 1.91 | 3.03 | 20230.92 |  |  |
| 1.91 | 3.71 | 13817.31 |  |  |
| 1.92 | 3.03 | 13493.97 |  |  |
| 1.92 | 1.9 | 26717.47 |  |  |
| 1.933 | 1.92 | 17887.14 |  |  |
| 1.946 | 1.93 | 13624.58 |  |  |
| 1.952 | 1.96 | 17393.22 |  |  |
| 1.958 | 1.96 | 17236.78 |  |  |
| 1.967 | 2.34 | 10744.68 |  |  |
| 1.969 | 1.99 | 38921.55 |  |  |
| 1.978 | 3.37 | 10916.93 |  |  |
| 1.978 | 1.98 | 55435.09 |  |  |
| 1.981 | 3.4 | 10474.91 |  |  |
| 1.985 | 1.99 | 56649.03 |  |  |
| 1.985 | 3.36 | 12802.41 |  |  |
| 1.986 | 2.32 | 14553.02 |  |  |
| 1.994 | 2 | 108332.97 |  |  |
| 2 | 2.01 | 88447.55 |  |  |
| 2.005 | 3.36 | 12298.92 |  |  |
| 2.011 | 3.37 | 11469.22 |  |  |
| 2.013 | 2.01 | 62024.28 |  |  |
| 2.013 | 2.35 | 19792.51 |  |  |
| 2.02 | 2.35 | 40043.46 |  |  |
| 2.02 | 3.73 | 14731.99 |  |  |
| 2.021 | 2.02 | 79277.76 |  |  |
| 2.027 | 2.02 | 102831.44 |  |  |
| 2.029 | 2.32 | 13784.24 |  |  |
| 2.037 | 2.04 | 88297.71 |  |  |
| 2.037 | 2.35 | 31750.35 |  |  |
| 2.038 | 3.73 | 23311.61 |  |  |
| 2.05 | 3.36 | 11814.48 |  |  |
| 2.054 | 2.46 | 15507.59 |  |  |
| 2.057 | 2.35 | 18365.43 |  |  |
| 2.058 | 3.72 | 20374.99 |  |  |
| 2.062 | 2.06 | 486852.1 |  |  |
| 2.067 | 3.35 | 12384.95 |  |  |
| 2.071 | 2.43 | 95599.86 |  |  |
| 2.072 | 3.72 | 34097.15 |  |  |
| 2.081 | 2.1 | 145227.05 |  |  |
| 2.089 | 2.1 | 208687.39 |  |  |
| 2.089 | 3.72 | 49779.14 |  |  |
| 2.09 | 2.43 | 94362.18 |  |  |
| 2.096 | 2.1 | 329298.64 | L_Glutamic_acid_1 |  |
| 2.096 | 3.73 | 29722.88 | clique_0002 | L_Glutamine_1 |
| 2.105 | 2.44 | 36704.15 | clique_0002 |  |
| 2.105 | 2.1 | 235666.61 |  |  |
| 2.107 | 3.73 | 39312.98 |  |  |
| 2.112 | 3.72 | 43253.1 |  |  |
| 2.112 | 2.1 | 360240.94 |  |  |
| 2.118 | 3.72 | 39609.02 |  |  |
| 2.118 | 2.43 | 124366.39 |  |  |
| 2.124 | 2.34 | 38279.14 |  |  |
| 2.127 | 2.11 | 258614.6 |  |  |
| 2.129 | 3.73 | 40791.88 |  |  |
| 2.129 | 2.43 | 67500.02 |  |  |
| 2.134 | 3.72 | 60106.79 |  |  |
| 2.134 | 2.43 | 135203.68 |  |  |
| 2.141 | 3.72 | 18304.07 |  |  |
| 2.146 | 2.14 | 88884.82 |  |  |
| 2.153 | 2.41 | 22201.84 |  |  |
| 2.155 | 2.13 | 49359.86 |  |  |
| 2.16 | 2.13 | 36364.08 |  |  |
| 2.162 | 2.39 | 13891.03 |  |  |
| 2.169 | 2.14 | 29444.49 |  |  |
| 2.17 | 2.42 | 15226.75 |  |  |
| 2.18 | 2.17 | 91953.63 |  |  |
| 2.192 | 2.19 | 56082.96 |  |  |
| 2.209 | 1 | 18316.21 |  |  |
| 2.209 | 2.21 | 88167.54 |  |  |
| 2.211 | 3.58 | 10378.51 |  |  |
| 2.219 | 2.22 | 160301.44 |  |  |
| 2.221 | 1 | 36515.72 |  |  |
| 2.226 | 1 | 29982.14 |  |  |
| 2.23 | 3.59 | 12936.51 |  |  |
| 2.232 | 2.23 | 54861.24 |  |  |
| 2.239 | 1 | 23502.97 |  |  |
| 2.24 | 2.25 | 52901.92 |  |  |
| 2.245 | 2.25 | 52519.5 | L_Valine_1 |  |
| 2.246 | 3.59 | 16678.18 | clique_0003 |  |
| 2.246 | 0.99 | 16803.68 | L_Valine_1 | 2_Ethylacrylic_acid_1 |
| 2.256 | 1 | 22300.5 | clique_0003 |  |
| 2.258 | 2.25 | 56722.38 |  |  |
| 2.264 | 1 | 17714.58 |  |  |
| 2.266 | 1.18 | 12260.66 |  |  |
| 2.267 | 2.27 | 114139.86 |  |  |
| 2.268 | 3.43 | 10392.23 |  |  |
| 2.275 | 2.26 | 54564.83 |  |  |
| 2.277 | 1 | 24340.73 |  |  |
| 2.281 | 1 | 31452.83 |  |  |
| 2.282 | 1.19 | 13865.12 |  |  |
| 2.284 | 2.28 | 307973.87 |  |  |
| 2.285 | 2.1 | 14357.66 |  |  |
| 2.291 | 3.59 | 10801.45 |  |  |
| 2.294 | 1 | 16517.89 |  |  |
| 2.298 | 1 | 11694.44 |  |  |
| 2.301 | 2.11 | 15685.71 |  |  |
| 2.302 | 2.31 | 43915.07 |  |  |
| 2.302 | 1.18 | 32309.94 |  |  |
| 2.308 | 2.05 | 23808.34 |  |  |
| 2.31 | 2.32 | 38503.5 |  |  |
| 2.318 | 1.18 | 31574.98 |  |  |
| 2.322 | 3.73 | 35239.76 |  |  |
| 2.323 | 2.08 | 93610.74 |  |  |
| 2.323 | 2.34 | 147198.78 |  |  |
| 2.331 | 2.34 | 41351.12 |  |  |
| 2.342 | 2.34 | 300304.26 |  |  |
| 2.342 | 3.73 | 30673.63 |  |  |
| 2.343 | 2.05 | 49873.15 |  |  |
| 2.358 | 2.54 | 12047.63 |  |  |
| 2.358 | 2.35 | 601015.73 |  |  |
| 2.363 | 2.05 | 51692.32 |  |  |
| 2.363 | 3.73 | 15956 |  |  |
| 2.364 | 1.18 | 32195.19 |  |  |
| 2.369 | 2.38 | 58436.87 |  |  |
| 2.374 | 2.11 | 19833.94 |  |  |
| 2.382 | 1.18 | 30290.69 |  |  |
| 2.387 | 2.04 | 23655.94 |  |  |
| 2.388 | 4.17 | 11624.58 |  |  |
| 2.388 | 2.39 | 153225.77 |  |  |
| 2.396 | 2.12 | 24122.99 |  |  |
| 2.4 | 1.18 | 12009.1 |  |  |
| 2.412 | 3.72 | 64436.11 |  |  |
| 2.412 | 2.43 | 231195.68 |  |  |
| 2.412 | 2.1 | 226713.65 |  |  |
| 2.413 | 3.41 | 25388.86 |  |  |
| 2.417 | 2.43 | 242736.79 |  |  |
| 2.418 | 1.18 | 13634.53 |  |  |
| 2.423 | 3.41 | 30412.15 |  |  |
| 2.428 | 3.41 | 28687.5 |  |  |
| 2.43 | 2.43 | 437435.75 |  |  |
| 2.431 | 2.11 | 111274.61 |  |  |
| 2.433 | 3.72 | 93037.93 | clique_0002 |  |
| 2.439 | 2.43 | 372746.48 | L_Glutamine_1 |  |
| 2.439 | 2.1 | 121199.82 | clique_0002 |  |
| 2.44 | 3.41 | 23737.65 |  |  |
| 2.45 | 2.43 | 247372.02 |  |  |
| 2.452 | 3.71 | 11541.18 |  |  |
| 2.457 | 2.1 | 154369.75 |  |  |
| 2.458 | 2.43 | 97462.95 |  |  |
| 2.458 | 3.72 | 31252 |  |  |
| 2.468 | 2.47 | 28771.93 |  |  |
| 2.474 | 2.07 | 14371.16 |  |  |
| 2.475 | 2.43 | 43942.35 |  |  |
| 2.484 | 2.5 | 40369.84 |  |  |
| 2.499 | 2.51 | 152665.6 |  |  |
| 2.503 | 2.65 | 471424.56 |  |  |
| 2.541 | 2.65 | 658031.56 |  |  |
| 2.542 | 2.54 | 248240.73 |  |  |
| 2.567 | 2.56 | 18307.13 |  |  |
| 2.576 | 2.57 | 209847.57 |  |  |
| 2.586 | 2.58 | 19156.86 |  |  |
| 2.588 | 0.92 | 10961.02 |  |  |
| 2.597 | 2.6 | 38807.78 |  |  |
| 2.605 | 0.92 | 11919.58 |  |  |
| 2.61 | 2.12 | 11065.02 |  |  |
| 2.61 | 2.63 | 33734.02 |  |  |
| 2.619 | 2.65 | 20822.28 |  |  |
| 2.629 | 2.11 | 11536.93 |  |  |
| 2.636 | 2.64 | 379968.93 |  |  |
| 2.641 | 2.53 | 635340.55 |  |  |
| 2.678 | 2.52 | 446325.15 |  |  |
| 2.682 | 2.66 | 129662.34 |  |  |
| 2.701 | 2.7 | 119138.34 |  |  |
| 2.711 | 2.71 | 73201.62 |  |  |
| 2.726 | 2.72 | 235639.22 |  |  |
| 2.74 | 2.73 | 17437.75 |  |  |
| 2.75 | 2.77 | 15720.14 |  |  |
| 2.753 | 2.74 | 16263.49 |  |  |
| 2.763 | 2.75 | 20707.58 |  |  |
| 2.771 | 2.77 | 28917.04 |  |  |
| 2.776 | 2.88 | 13800.19 |  |  |
| 2.783 | 2.78 | 40733.23 |  |  |
| 2.796 | 2.8 | 24182.48 |  |  |
| 2.816 | 2.8 | 33494.09 |  |  |
| 2.848 | 2.85 | 169382.31 |  |  |
| 2.849 | 7.91 | 11383.29 |  |  |
| 2.869 | 2.88 | 22073.74 | DSS_1 |  |
| 2.884 | 0.62 | 36189.47 | clique_0004 |  |
| 2.884 | 2.89 | 45129.47 |  |  |
| 2.884 | 1.75 | 35327.93 |  |  |
| 2.894 | 2.9 | 32264.08 | DSS_1 |  |
| 2.905 | 1.75 | 30246.23 | clique_0004 |  |
| 2.905 | 0.63 | 32287.24 |  |  |
| 2.905 | 2.9 | 50947.69 |  |  |
| 2.925 | 2.9 | 38406.25 |  |  |
| 2.925 | 1.75 | 31690.83 |  |  |
| 2.926 | 0.63 | 33432.17 |  |  |
| 2.936 | 2.91 | 26202.04 |  |  |
| 2.95 | 2.95 | 12640.6 |  |  |
| 2.957 | 2.96 | 13286.56 |  |  |
| 2.97 | 2.97 | 18563.4 |  |  |
| 2.996 | 3.01 | 106634.26 |  |  |
| 2.998 | 1.71 | 47797.15 |  |  |
| 2.998 | 1.87 | 42798.6 |  |  |
| 2.998 | 1.46 | 40061.54 |  |  |
| 2.998 | 3.72 | 13647.09 |  |  |
| 3.005 | 3 | 222383.04 |  |  |
| 3.017 | 3.02 | 131250.85 |  |  |
| 3.018 | 1.46 | 43139.11 | clique_0007 | N_alpha__Acetyl_ornithine_1 |
| 3.018 | 1.71 | 74679.57 | clique_0005 | Betaine_1 |
| 3.019 | 1.88 | 53494.99 | clique_0005 | clique_0006 |
| 3.022 | 3.72 | 19620.84 | clique_0006 | L_Glutamic_acid_1 |
| 3.026 | 3.02 | 392570.96 |  |  |
| 3.027 | 3.91 | 16627.56 |  |  |
| 3.031 | 4.03 | 11023.53 |  |  |
| 3.037 | 1.46 | 37401.99 |  |  |
| 3.038 | 1.72 | 53541.89 |  |  |
| 3.039 | 3.03 | 115478.99 |  |  |
| 3.039 | 3.72 | 18067.97 |  |  |
| 3.039 | 1.88 | 67162.74 |  |  |
| 3.048 | 3.03 | 57458.56 |  |  |
| 3.053 | 3.93 | 10448.73 |  |  |
| 3.059 | 3.71 | 14636.72 |  |  |
| 3.06 | 3.05 | 31111.28 |  |  |
| 3.06 | 1.87 | 28448.09 |  |  |
| 3.066 | 3.07 | 28897.51 |  |  |
| 3.074 | 3.08 | 38787.57 |  |  |
| 3.093 | 3.27 | 13897.04 |  |  |
| 3.094 | 3.1 | 174078.29 |  |  |
| 3.094 | 3.97 | 26505.37 |  |  |
| 3.121 | 3.12 | 285286.73 |  |  |
| 3.123 | 3.96 | 11565.97 |  |  |
| 3.132 | 3.98 | 27111.65 |  |  |
| 3.138 | 3.14 | 115087.72 |  |  |
| 3.152 | 3.14 | 93621.65 |  |  |
| 3.164 | 3.17 | 56158.59 |  |  |
| 3.177 | 3.18 | 243181.16 |  |  |
| 3.185 | 3.18 | 338166.67 |  |  |
| 3.21 | 3.21 | 1376627.5 |  |  |
| 3.21 | 3.03 | 17266.95 |  |  |
| 3.211 | 4.62 | 178485.88 |  |  |
| 3.211 | 3.46 | 400822.81 |  |  |
| 3.212 | 3.71 | 136277.84 |  |  |
| 3.213 | 1.68 | 13809.6 |  |  |
| 3.213 | 1.87 | 27099.97 | clique_0009 |  |
| 3.213 | 3.89 | 119726.27 |  |  |
| 3.225 | 3.38 | 56211.1 |  |  |
| 3.231 | 1.88 | 34643.68 |  |  |
| 3.231 | 1.68 | 30048.72 |  |  |
| 3.231 | 3.04 | 10688.52 |  |  |
| 3.233 | 3.71 | 242433.55 | clique_0008 | clique_0009 |
| 3.233 | 3.89 | 238600.78 | clique_0001 | Taurine_1 |
| 3.234 | 3.43 | 915512.81 | clique_0001 | clique_0008 |
| 3.234 | 3.23 | 659292.34 |  |  |
| 3.235 | 4.63 | 387039.91 |  |  |
| 3.236 | 4.8 | 14534.1 |  |  |
| 3.248 | 3.07 | 11834.05 |  |  |
| 3.248 | 3.25 | 743519.29 |  |  |
| 3.249 | 1.88 | 22757.74 |  |  |
| 3.249 | 1.68 | 16150.67 |  |  |
| 3.254 | 3.89 | 135724.07 |  |  |
| 3.254 | 3.71 | 145256.8 |  |  |
| 3.254 | 4.63 | 338182.67 |  |  |
| 3.255 | 3.24 | 629450.15 |  |  |
| 3.255 | 3.45 | 730201.53 |  |  |
| 3.256 | 4.79 | 11520.5 |  |  |
| 3.262 | 3.96 | 40925.23 |  |  |
| 3.396 | 5.22 | 16972.83 |  |  |
| 3.401 | 2 | 18112.47 |  |  |
| 3.402 | 3.41 | 339324.11 |  |  |
| 3.403 | 3.51 | 290839.23 |  |  |
| 3.403 | 3.79 | 540504.4 |  |  |
| 3.403 | 3.24 | 94673.02 |  |  |
| 3.405 | 2.42 | 40551.2 |  |  |
| 3.407 | 5.22 | 28389.67 |  |  |
| 3.412 | 3.04 | 11736.34 |  |  |
| 3.412 | 2.01 | 10270.96 |  |  |
| 3.412 | 2.19 | 13320.18 |  |  |
| 3.412 | 0.09 | 13659.61 |  |  |
| 3.412 | 0.31 | 13597.14 |  |  |
| 3.413 | 3.23 | 403712.97 |  |  |
| 3.413 | 4.44 | 11779.83 |  |  |
| 3.414 | 2.42 | 26413.22 |  |  |
| 3.415 | 4.63 | 676812.64 |  |  |
| 3.415 | 3.72 | 545798.86 |  |  |
| 3.415 | 3.45 | 1329236.29 |  |  |
| 3.415 | 3.89 | 476565.54 |  |  |
| 3.416 | 4.8 | 23512.75 |  |  |
| 3.416 | 5.22 | 17447.36 |  |  |
| 3.417 | 5.59 | 10397.37 |  |  |
| 3.417 | 4.08 | 12858.25 |  |  |
| 3.421 | 2.42 | 18946.2 |  |  |
| 3.427 | 5.22 | 85791.71 |  |  |
| 3.427 | 3.72 | 465247.22 | Taurine_1 |  |
| 3.43 | 3.24 | 109445.19 | clique_0008 |  |
| 3.431 | 3.43 | 535747.36 |  |  |
| 3.431 | 4.63 | 263777.47 |  |  |
| 3.432 | 3.88 | 290883.89 | clique_0011 |  |
| 3.436 | 3.23 | 127147.1 | clique_0001 |  |
| 3.437 | 3.44 | 626257.67 |  |  |
| 3.437 | 4.63 | 280926.65 |  |  |
| 3.438 | 3.71 | 409137.4 | clique_0008 | clique_0011 |
| 3.439 | 4.8 | 10948.94 |  |  |
| 3.444 | 3.71 | 403326.17 |  |  |
| 3.445 | 3.23 | 116952.08 |  |  |
| 3.446 | 3.44 | 557732.02 |  |  |
| 3.446 | 4.63 | 268530.59 |  |  |
| 3.448 | 3.89 | 330162.37 | clique_0001 |  |
| 3.449 | 3.71 | 293823.18 |  |  |
| 3.452 | 3.44 | 648138.36 |  |  |
| 3.452 | 3.23 | 136245.72 |  |  |
| 3.454 | 4.07 | 11315.45 |  |  |
| 3.454 | 4.8 | 17608.49 |  |  |
| 3.457 | 4.63 | 722249.92 |  |  |
| 3.458 | 3.42 | 1063725.43 |  |  |
| 3.458 | 3.23 | 535427.44 |  |  |
| 3.458 | 3.72 | 298628.08 |  |  |
| 3.458 | 3.88 | 398827.32 |  |  |
| 3.459 | 4.8 | 29017.72 |  |  |
| 3.469 | 4.63 | 157242.66 |  |  |
| 3.469 | 3.71 | 237647.15 |  |  |
| 3.47 | 3.45 | 206115.58 |  |  |
| 3.471 | 3.24 | 43479.96 |  |  |
| 3.474 | 3.71 | 216992.59 |  |  |
| 3.474 | 3.89 | 155961.45 |  |  |
| 3.479 | 0.31 | 11555.56 |  |  |
| 3.479 | 3.05 | 12229.5 |  |  |
| 3.479 | 4.44 | 12607.42 |  |  |
| 3.481 | 3.23 | 565258.3 |  |  |
| 3.481 | 4.63 | 817085.32 |  |  |
| 3.481 | 3.44 | 1396534.4 |  |  |
| 3.481 | 3.89 | 152367.99 |  |  |
| 3.481 | 3.71 | 152847.25 |  |  |
| 3.482 | 4.8 | 26903 |  |  |
| 3.483 | 5.55 | 10880.06 |  |  |
| 3.484 | 5.2 | 11365.48 |  |  |
| 3.49 | 5.21 | 10721.22 |  |  |
| 3.503 | 3.23 | 217709.96 |  |  |
| 3.504 | 3.41 | 274427.83 |  |  |
| 3.504 | 4.63 | 236746.97 |  |  |
| 3.504 | 4.07 | 11118.29 |  |  |
| 3.505 | 4.79 | 10709.33 |  |  |
| 3.506 | 5.22 | 104948.85 |  |  |
| 3.507 | 3.53 | 275033.77 |  |  |
| 3.507 | 3.8 | 376279.12 | Choline_1 |  |
| 3.513 | 4.06 | 13255.35 | small_0003 |  |
| 3.516 | 5.22 | 79595.3 | small_0006 |  |
| 3.516 | 3.52 | 273069.66 |  |  |
| 3.517 | 3.8 | 373743.86 |  |  |
| 3.518 | 3.41 | 93953.5 |  |  |
| 3.526 | 4.28 | 11956.59 |  |  |
| 3.527 | 2.4 | 10345.42 |  |  |
| 3.527 | 8.4 | 12170.97 |  |  |
| 3.527 | 2.94 | 17743.46 |  |  |
| 3.528 | 1.98 | 12080.76 |  |  |
| 3.528 | 3.23 | 16977.27 |  |  |
| 3.529 | 3.57 | 1937211.24 |  |  |
| 3.531 | 1.3 | 52719.61 |  |  |
| 3.531 | 7.62 | 16499.93 |  |  |
| 3.577 | 5.02 | 20342.85 |  |  |
| 3.577 | 4.47 | 11846.74 |  |  |
| 3.577 | 3.96 | 44610.98 |  |  |
| 3.581 | 3.38 | 11209.79 | L_Valine_1 |  |
| 3.581 | 1 | 120168.13 | clique_0003 |  |
| 3.581 | 2.25 | 25418.57 |  |  |
| 3.587 | 3.38 | 17047.61 |  |  |
| 3.589 | 3.97 | 17873.12 | L_Valine_1 |  |
| 3.591 | 2.25 | 27822.63 | clique_0003 |  |
| 3.591 | 0.99 | 123273.68 |  |  |
| 3.592 | 3.27 | 33311.48 |  |  |
| 3.593 | 3.39 | 18936.39 |  |  |
| 3.593 | 4.03 | 13419.61 |  |  |
| 3.593 | 3.59 | 441429.95 |  |  |
| 3.614 | 3.38 | 18416.35 |  |  |
| 3.616 | 3.27 | 27616.8 |  |  |
| 3.619 | 1.17 | 156884.39 |  |  |
| 3.623 | 2.59 | 16464.37 |  |  |
| 3.623 | 2.45 | 12199.13 |  |  |
| 3.623 | 4.28 | 23410.14 |  |  |
| 3.623 | 2.27 | 12863.58 |  |  |
| 3.623 | 6.95 | 15852.56 |  |  |
| 3.623 | 2.94 | 34076.37 |  |  |
| 3.623 | 6.73 | 19765.04 |  |  |
| 3.623 | 3.25 | 33964.74 |  |  |
| 3.623 | 0.55 | 16074.7 |  |  |
| 3.623 | 3.79 | 151394.07 |  |  |
| 3.623 | 8.49 | 16314.41 |  |  |
| 3.623 | 9.47 | 11222.26 |  |  |
| 3.624 | 8.4 | 15828.3 |  |  |
| 3.624 | 7.27 | 20534.12 |  |  |
| 3.624 | -0.01 | 16448.97 |  |  |
| 3.624 | 0.27 | 22514.14 |  |  |
| 3.624 | 2.03 | 20249.68 |  |  |
| 3.625 | 3.36 | 35548.45 |  |  |
| 3.626 | 3.61 | 3966726.34 |  |  |
| 3.626 | 8.03 | 13616.57 |  |  |
| 3.627 | 5.75 | 29244.71 |  |  |
| 3.627 | 7.81 | 12614.55 |  |  |
| 3.628 | 10.43 | 11584.87 |  |  |
| 3.628 | 7.57 | 27928.6 |  |  |
| 3.628 | 6.53 | 15419.67 |  |  |
| 3.628 | 1.48 | 13401.79 |  |  |
| 3.628 | 7.12 | 13622.43 |  |  |
| 3.628 | -0.43 | 17887.07 |  |  |
| 3.628 | 4.73 | 31510.05 |  |  |
| 3.685 | 4.74 | 10750.98 |  |  |
| 3.689 | 4 | 15862.96 |  |  |
| 3.69 | 2.1 | 15715.51 |  |  |
| 3.692 | 3.71 | 950688.53 |  |  |
| 3.693 | 3.44 | 538804.74 |  |  |
| 3.693 | 3.24 | 79191.48 | clique_0008 |  |
| 3.693 | 4.63 | 96127.42 |  |  |
| 3.694 | 3.88 | 186047.05 |  |  |
| 3.695 | 4.78 | 10418.42 |  |  |
| 3.696 | 1.7 | 32075.52 |  |  |
| 3.696 | 0.94 | 34847.82 |  |  |
| 3.702 | 4.63 | 26292.24 |  |  |
| 3.703 | 1.88 | 60775.82 | clique_0009 | clique_0010 |
| 3.704 | 3.03 | 48118.93 |  |  |
| 3.704 | 1.46 | 31011.92 |  |  |
| 3.704 | 0.09 | 10486.77 |  |  |
| 3.704 | 1.72 | 30013.94 |  |  |
| 3.705 | 3.23 | 91063.62 | clique_0009 |  |
| 3.705 | 4.01 | 15126.08 |  |  |
| 3.706 | 5.22 | 293040.86 | small_0007 |  |
| 3.706 | 3.43 | 925943.44 | clique_0008 | clique_0011 |
| 3.707 | 3.71 | 1408573.32 | 2_Aminobutyric_acid_1 |  |
| 3.708 | 0.95 | 16750.34 | small_0008 |  |
| 3.708 | 10.59 | 10282.25 |  |  |
| 3.708 | 4.76 | 10702.41 |  |  |
| 3.709 | 2.1 | 137064.52 |  |  |
| 3.71 | 1.65 | 22656.51 |  |  |
| 3.71 | 2.43 | 125228.62 |  |  |
| 3.719 | 2.33 | 40250.53 | alpha,epsilon_  Diaminopimelic_acid_2 | Tetrahydrofuran_1 |
| 3.719 | 1.88 | 95864.27 | clique_0006 |  |
| 3.72 | 3.03 | 40666.04 | clique_0006 |  |
| 3.72 | 1.46 | 32146.65 |  |  |
| 3.721 | 1.71 | 39790.2 | clique_0010 |  |
| 3.721 | 0.3 | 10482.59 |  |  |
| 3.722 | 2.76 | 10669.93 |  |  |
| 3.722 | 0.94 | 17497.6 |  |  |
| 3.722 | 0.1 | 11063.8 |  |  |
| 3.722 | 3.88 | 309317.46 | clique_0011 |  |
| 3.723 | 3.72 | 1656572.91 |  |  |
| 3.724 | 3.44 | 746223.76 |  |  |
| 3.725 | 3.23 | 113196.01 |  |  |
| 3.725 | 4.63 | 118076.12 | L_Glutamic_acid_1 |  |
| 3.725 | 2.1 | 138316.69 | clique_0002 |  |
| 3.726 | 2.43 | 123068.82 | clique_0002 |  |
| 3.729 | 3.41 | 476539.96 |  |  |
| 3.729 | 5.22 | 135076.03 |  |  |
| 3.73 | 0.94 | 31829.87 |  |  |
| 3.734 | 4.63 | 52304.53 |  |  |
| 3.734 | 3.02 | 39719.46 | L_Glutamic_acid_1 |  |
| 3.735 | 1.71 | 43666.38 | L_Glutamic_acid_1 |  |
| 3.736 | 1.88 | 45973.53 | L_Glutamic_acid_1 |  |
| 3.736 | 1.46 | 38372.23 | L_Glutamic_acid_1 |  |
| 3.736 | 3.23 | 110486.16 |  |  |
| 3.736 | 3.88 | 393389.76 |  |  |
| 3.737 | 2.33 | 16573.42 |  |  |
| 3.737 | 3.44 | 709260.45 |  |  |
| 3.738 | 3.72 | 1287232.63 |  |  |
| 3.741 | 2.43 | 102468.66 |  |  |
| 3.741 | 1.55 | 17399.91 |  |  |
| 3.742 | 1.38 | 11501.9 |  |  |
| 3.742 | 2.1 | 107686.42 |  |  |
| 3.743 | 5.23 | 11261.22 |  |  |
| 3.744 | 3.75 | 1123356.72 |  |  |
| 3.749 | 2.34 | 37082.59 |  |  |
| 3.75 | 3.65 | 521451.41 |  |  |
| 3.75 | 2.08 | 55273.62 |  |  |
| 3.758 | 1.46 | 237106.09 |  |  |
| 3.759 | 3.58 | 899351.94 |  |  |
| 3.761 | 3.77 | 1270031.59 |  |  |
| 3.761 | 3.4 | 135607.5 |  |  |
| 3.762 | 4.74 | 11450.64 |  |  |
| 3.763 | 5.22 | 16014.75 |  |  |
| 3.766 | 4.74 | 11598.11 |  |  |
| 3.772 | 5.22 | 25674.46 |  |  |
| 3.774 | 2.11 | 10761.95 |  |  |
| 3.776 | 3.77 | 2155271.31 |  |  |
| 3.776 | 3.56 | 720504.6 |  |  |
| 3.777 | 1.46 | 236581.01 |  |  |
| 3.778 | 4.92 | 12784.73 |  |  |
| 3.778 | 7.62 | 11059.29 |  |  |
| 3.778 | 7.45 | 14130.05 |  |  |
| 3.778 | 3.93 | 30495.79 |  |  |
| 3.778 | 4.73 | 16960.24 |  |  |
| 3.782 | 4.74 | 15073.5 |  |  |
| 3.785 | 3.41 | 31802.74 |  |  |
| 3.788 | 3.63 | 577557.15 |  |  |
| 3.788 | 3.93 | 68407.68 |  |  |
| 3.792 | 3.57 | 668957.44 |  |  |
| 3.797 | 1.45 | 17879.48 |  |  |
| 3.797 | 3.79 | 478198.28 |  |  |
| 3.797 | 5.22 | 26397.42 |  |  |
| 3.798 | 4.74 | 10786.12 |  |  |
| 3.801 | 3.57 | 292907.73 |  |  |
| 3.801 | 3.4 | 131214.81 |  |  |
| 3.802 | 5.22 | 27093.77 |  |  |
| 3.804 | 3.76 | 482641.13 |  |  |
| 3.811 | 3.14 | 10952.35 |  |  |
| 3.814 | 3.82 | 765797.13 |  |  |
| 3.814 | 3.41 | 174894.88 |  |  |
| 3.821 | 3.41 | 307856.78 |  |  |
| 3.821 | 5.22 | 57133.89 |  |  |
| 3.821 | 3.51 | 285878.75 |  |  |
| 3.822 | 3.81 | 1409044.53 |  |  |
| 3.823 | 4.01 | 13336.24 |  |  |
| 3.835 | 5.22 | 18020.33 |  |  |
| 3.835 | 3.77 | 388212.91 |  |  |
| 3.836 | 3.52 | 94396.43 |  |  |
| 3.84 | 5.22 | 11460.88 |  |  |
| 3.848 | 3.83 | 760506.02 |  |  |
| 3.849 | 3.41 | 99779.12 |  |  |
| 3.849 | 5.23 | 11491.35 |  |  |
| 3.849 | 3.52 | 136150.58 |  |  |
| 3.854 | 3.83 | 666172.51 |  |  |
| 3.855 | 3.64 | 32025.08 |  |  |
| 3.871 | 3.89 | 1163088.36 |  |  |
| 3.872 | 3.71 | 429534.31 |  |  |
| 3.873 | 3.44 | 635338.89 |  |  |
| 3.873 | 4.07 | 11311.18 |  |  |
| 3.874 | 4.63 | 80154.73 |  |  |
| 3.876 | 3.23 | 158166.23 |  |  |
| 3.876 | 3.44 | 672257.73 | clique_0011 |  |
| 3.877 | 3.89 | 1294886.3 |  |  |
| 3.879 | 4.08 | 19861.14 |  |  |
| 3.879 | 3.71 | 264633.92 | clique_0011 |  |
| 3.888 | 3.28 | 10646.07 |  |  |
| 3.889 | 3.94 | 49766.84 |  |  |
| 3.898 | 3.72 | 127806.02 |  |  |
| 3.902 | 4.63 | 41415.56 |  |  |
| 3.902 | 3.89 | 1020693.71 |  |  |
| 3.903 | 3.44 | 336916.61 | clique_0001 | Betaine_1 |
| 3.903 | 3.23 | 73222.04 | clique_0001 |  |
| 3.907 | 3.72 | 119959.87 |  |  |
| 3.907 | 3.44 | 321275.77 |  |  |
| 3.908 | 3.89 | 929146.94 |  |  |
| 3.911 | 3.6 | 19593.69 |  |  |
| 3.914 | 3.9 | 404003.13 |  |  |
| 3.919 | 3.6 | 11842.91 |  |  |
| 3.923 | 3.08 | 20223.15 |  |  |
| 3.924 | 1.32 | 11337.98 |  |  |
| 3.925 | 3.92 | 175833.76 |  |  |
| 3.926 | 3.62 | 11372.14 |  |  |
| 3.929 | 3.61 | 11372.23 |  |  |
| 3.937 | 3.94 | 182068.5 |  |  |
| 3.938 | 3.2 | 14595.71 |  |  |
| 3.94 | 3.61 | 10657.2 |  |  |
| 3.946 | 3.08 | 15201.13 |  |  |
| 3.947 | 3.31 | 12614.77 |  |  |
| 3.947 | 3.59 | 11744.87 |  |  |
| 3.948 | 3.94 | 145171.56 |  |  |
| 3.956 | 3.95 | 122361.62 |  |  |
| 3.958 | 3.81 | 30308.39 |  |  |
| 3.959 | 3.11 | 32147.46 |  |  |
| 3.961 | 3.27 | 20372.84 |  |  |
| 3.962 | 3.6 | 12741.7 |  |  |
| 3.966 | 3.18 | 11409.68 |  |  |
| 3.967 | 3.05 | 11518.85 |  |  |
| 3.969 | 0.88 | 17348.13 |  |  |
| 3.97 | 3.97 | 101209.29 |  |  |
| 3.972 | 3.08 | 10944.95 |  |  |
| 3.976 | 3.59 | 12257.79 |  |  |
| 3.977 | 3.98 | 99299.26 |  |  |
| 3.978 | 3.29 | 22906.08 |  |  |
| 3.986 | 0.88 | 14015.5 |  |  |
| 3.987 | 3.97 | 70536.56 |  |  |
| 3.99 | 3.24 | 23118.61 |  |  |
| 3.997 | 3.99 | 48611.65 |  |  |
| 3.998 | 0.88 | 16754.16 |  |  |
| 4 | 3.12 | 23924.96 |  |  |
| 4.002 | 3.28 | 10869.7 |  |  |
| 4.004 | 4 | 30482.15 |  |  |
| 4.018 | 4.02 | 22656.6 |  |  |
| 4.041 | 4.04 | 139672.73 |  |  |
| 4.049 | 3.53 | 15072.44 | Choline_1 |  |
| 4.055 | 3.5 | 12682 | small_0003 |  |
| 4.055 | 4.06 | 54525.38 |  |  |
| 4.069 | 1.32 | 50741.99 |  |  |
| 4.072 | 4.09 | 308266.88 |  |  |
| 4.074 | 1.41 | 28804.35 |  |  |
| 4.075 | 1.21 | 20866.22 |  |  |
| 4.088 | 1.5 | 10617.95 |  |  |
| 4.09 | 1.31 | 957475.16 |  |  |
| 4.091 | 4.1 | 384076.04 |  |  |
| 4.095 | 2 | 12632.46 |  |  |
| 4.107 | 4.1 | 384387.62 |  |  |
| 4.672 | 9.4 | 14627.41 |  |  |
| 4.672 | 5.72 | 12256.16 |  |  |
| 4.673 | 6.71 | 24343.76 |  |  |
| 4.673 | 9.76 | 11041.61 |  |  |
| 4.673 | 8.49 | 14582.98 |  |  |
| 4.674 | 0.18 | 13849.59 |  |  |
| 4.675 | 8.95 | 10763.49 |  |  |
| 4.676 | 6.37 | 21694.12 |  |  |
| 4.677 | -0.67 | 13586.42 |  |  |
| 4.677 | 5.34 | 27889.1 |  |  |
| 4.679 | 5.14 | 44471.18 |  |  |
| 4.68 | 7.12 | 28918.1 |  |  |
| 4.68 | 2.51 | 16776.75 |  |  |
| 4.681 | 7.95 | 38503.78 |  |  |
| 4.681 | 0.2 | 10498.35 |  |  |
| 4.681 | 5.7 | 45351 |  |  |
| 4.681 | 10.53 | 35593.13 |  |  |
| 4.681 | 8.97 | 28050.58 |  |  |
| 4.681 | 8.85 | 23054.17 |  |  |
| 4.681 | 6.03 | 13764.13 |  |  |
| 4.682 | 6.75 | 51563.81 |  |  |
| 4.682 | 2.94 | 25227.53 |  |  |
| 4.682 | 0.93 | 25594.12 |  |  |
| 4.683 | 5.9 | 15401.24 |  |  |
| 4.683 | 2.16 | 48311.66 |  |  |
| 4.683 | 9.27 | 52276.07 |  |  |
| 4.683 | 1.61 | 33881.83 |  |  |
| 4.684 | -0.75 | 14829.47 |  |  |
| 4.684 | 1.13 | 12798.02 |  |  |
| 4.684 | 9.98 | 36615.14 |  |  |
| 4.684 | 1.99 | 14685.87 |  |  |
| 4.684 | 9.1 | 60181.63 |  |  |
| 4.684 | 10.43 | 49966.5 |  |  |
| 4.684 | 3.85 | 16911.92 |  |  |
| 4.684 | 9.48 | 49069.83 |  |  |
| 4.684 | 8.1 | 40808.86 |  |  |
| 4.685 | 7.29 | 39929.81 |  |  |
| 4.685 | 5.58 | 77844.28 |  |  |
| 4.686 | 1.43 | 15146.87 |  |  |
| 4.686 | 5.75 | 42245.45 | small_0002 |  |
| 4.686 | 8.67 | 95935.86 |  |  |
| 4.687 | 8.23 | 72121.82 |  |  |
| 4.687 | 9.83 | 101428.66 |  |  |
| 4.687 | 7.73 | 20083.55 |  |  |
| 4.687 | 5.35 | 102089.91 |  |  |
| 4.687 | 5.02 | 236338.88 |  |  |
| 4.841 | 9.99 | 19368.84 |  |  |
| 4.841 | 10.3 | 19876.12 |  |  |
| 4.841 | 0.72 | 14629.99 |  |  |
| 4.841 | 7 | 13187.52 |  |  |
| 4.841 | 7.5 | 20175.02 |  |  |
| 4.842 | 6.66 | 16393.48 |  |  |
| 4.842 | 9.76 | 18801.22 |  |  |
| 4.842 | 8.07 | 19033.16 |  |  |
| 4.842 | 2.56 | 13161.56 |  |  |
| 4.842 | 2.91 | 14228.97 |  |  |
| 4.842 | 1.2 | 13315.69 |  |  |
| 4.842 | 8.49 | 16398.1 |  |  |
| 4.842 | 1.76 | 11083.5 |  |  |
| 4.843 | 7.84 | 11186.93 |  |  |
| 4.843 | 3.1 | 12439.12 |  |  |
| 4.843 | 4.61 | 22769.75 |  |  |
| 4.844 | 4.25 | 13836.15 |  |  |
| 4.844 | 3.39 | 16403.9 |  |  |
| 4.845 | -0.09 | 10748.69 |  |  |
| 4.848 | 4.62 | 14747.8 |  |  |
| 4.853 | 4.61 | 12503.57 |  |  |
| 4.859 | 4.61 | 12934.38 |  |  |
| 4.867 | 4.62 | 12611.43 |  |  |
| 4.872 | 4.62 | 14750.06 |  |  |
| 4.881 | 4.61 | 13216.67 |  |  |
| 4.884 | 4.62 | 14260.36 |  |  |
| 4.907 | 4.61 | 12814.08 |  |  |
| 4.914 | 4.61 | 10990.37 |  |  |
| 4.941 | 4.62 | 11577.59 |  |  |
| 4.95 | 4.61 | 10669.59 |  |  |
| 5.17 | 5.17 | 12366.92 |  |  |
| 5.174 | 5.17 | 10608.99 |  |  |
| 5.218 | 5.22 | 464255.81 |  |  |
| 5.218 | 3.52 | 101985.79 | small_0006 |  |
| 5.218 | 3.71 | 102942.24 | small_0007 |  |
| 5.227 | 3.71 | 81308.63 |  |  |
| 5.227 | 3.52 | 84200.42 |  |  |
| 5.228 | 5.22 | 525372.61 |  |  |
| 5.428 | 5.43 | 13636.84 |  |  |
| 5.436 | 4.69 | 10932.29 |  |  |
| 5.447 | 4.7 | 13096.78 |  |  |
| 5.461 | 4.7 | 13854.62 |  |  |
| 5.472 | 4.69 | 17444.04 |  |  |
| 5.477 | 4.69 | 16732.47 |  |  |
| 5.486 | 4.7 | 18234.42 |  |  |
| 5.497 | 4.69 | 20489.8 |  |  |
| 5.506 | 5.51 | 11949.48 |  |  |
| 5.507 | 4.7 | 23740.12 |  |  |
| 5.513 | 4.7 | 24444.51 |  |  |
| 5.52 | 4.7 | 24784.88 |  |  |
| 5.523 | 5.52 | 147324.91 |  |  |
| 5.529 | 4.7 | 29625.4 |  |  |
| 5.541 | 5.76 | 15528.01 |  |  |
| 5.543 | 4.69 | 35732.29 |  |  |
| 5.549 | 5.73 | 13157.47 |  |  |
| 5.549 | 4.7 | 39282.79 |  |  |
| 5.554 | 5.73 | 15870.29 |  |  |
| 5.562 | 5.74 | 16851.68 |  |  |
| 5.562 | 4.7 | 43544.72 |  |  |
| 5.567 | 5.74 | 18843.99 |  |  |
| 5.575 | 4.69 | 50707.86 |  |  |
| 5.577 | 5.74 | 19046.73 |  |  |
| 5.582 | 4.7 | 53801.57 |  |  |
| 5.583 | 5.74 | 22886.57 |  |  |
| 5.587 | 5.75 | 21400.36 |  |  |
| 5.593 | 4.69 | 58128.89 |  |  |
| 5.596 | 5.74 | 26085.04 |  |  |
| 5.601 | 5.74 | 27089.88 |  |  |
| 5.601 | 4.69 | 66747.06 |  |  |
| 5.606 | 5.75 | 26842.52 |  |  |
| 5.617 | 5.74 | 32283.67 |  |  |
| 5.631 | 4.69 | 98212.5 |  |  |
| 5.631 | 5.74 | 38716.48 |  |  |
| 5.639 | 4.69 | 104138.62 |  |  |
| 5.639 | 5.74 | 40334.24 |  |  |
| 5.646 | 5.09 | 27862.77 |  |  |
| 5.657 | 5.09 | 32875.13 |  |  |
| 5.659 | 5.73 | 50364.98 |  |  |
| 5.669 | 5.74 | 56878.53 |  |  |
| 5.727 | 5.74 | 102959.89 |  |  |
| 5.732 | 4.69 | 260437.64 |  |  |
| 5.737 | 5.74 | 102325.63 |  |  |
| 5.74 | 4.69 | 263874.63 |  |  |
| 5.742 | 5.74 | 103670.14 |  |  |
| 5.745 | 4.69 | 262810.08 | small_0002 |  |
| 5.746 | 5.74 | 103496.97 |  |  |
| 5.757 | 5.74 | 98295.9 |  |  |
| 5.763 | 5.74 | 94773.47 |  |  |
| 5.77 | 5.74 | 88198.7 |  |  |
| 5.796 | 5.74 | 66864.12 |  |  |
| 5.805 | 5.74 | 63175.84 |  |  |
| 5.821 | 5.75 | 50975.76 |  |  |
| 6.839 | 4.67 | 11260.87 |  |  |
| 6.846 | 4.68 | 11705.58 |  |  |
| 6.857 | 7.18 | 10773.09 |  |  |
| 6.859 | 4.67 | 10321.91 |  |  |
| 6.862 | 7.18 | 11343.62 |  |  |
| 6.87 | 7.18 | 71064.02 |  |  |
| 6.874 | 6.88 | 60251.89 |  |  |
| 6.892 | 6.88 | 76893.51 |  |  |
| 6.894 | 7.18 | 82719.79 |  |  |
| 7.03 | 7.74 | 17853.32 | small_0001 |  |
| 7.031 | 7.03 | 73644.57 |  |  |
| 7.168 | 6.88 | 66400.52 |  |  |
| 7.17 | 7.18 | 79157.47 |  |  |
| 7.189 | 7.18 | 68797.28 |  |  |
| 7.192 | 6.88 | 56982.87 |  |  |
| 7.274 | 7.22 | 11283.83 |  |  |
| 7.311 | 7.32 | 76102.25 |  |  |
| 7.326 | 7.33 | 69781.45 |  |  |
| 7.361 | 7.36 | 26355.28 |  |  |
| 7.367 | 7.57 | 19325.55 |  |  |
| 7.371 | 7.38 | 25607.41 |  |  |
| 7.379 | 7.38 | 35157.82 |  |  |
| 7.387 | 7.37 | 30453.41 | 4_Aminoantipyrine_1 |  |
| 7.39 | 7.58 | 19953.63 | small_0004 | small_0005 |
| 7.4 | 7.34 | 56294.99 |  |  |
| 7.419 | 7.4 | 65867.74 |  |  |
| 7.437 | 7.33 | 17569.47 |  |  |
| 7.524 | 7.73 | 10964.12 |  |  |
| 7.542 | 7.56 | 14125.29 |  |  |
| 7.559 | 7.6 | 13499.58 |  |  |
| 7.564 | 7.4 | 10537.21 | small_0004 |  |
| 7.58 | 7.58 | 19437.22 | 4_Aminoantipyrine_1 |  |
| 7.581 | 7.38 | 16381.8 | small_0005 |  |
| 7.599 | 7.59 | 33165.5 |  |  |
| 7.611 | 7.6 | 12346.9 |  |  |
| 7.617 | 7.6 | 15235.42 |  |  |
| 7.623 | 7.6 | 10920.18 |  |  |
| 7.659 | 7.66 | 10520.42 |  |  |
| 7.738 | 7.03 | 16800.38 | small_0001 |  |
| 7.738 | 7.74 | 64347.34 |  |  |
| 7.923 | 7.92 | 23710.68 |  |  |
| 8.166 | 8.16 | 18728.35 |  |  |
| 8.177 | 8.18 | 16417.09 |  |  |
| 8.443 | 8.44 | 82438.03 |  |  |
| 9.267 | 9.27 | 35327.6 |  |  |

**Supplementary table 3. Metabolites assigned by NMR metabolomics.** The table shows the assigned metabolites, the proton chemical shift values in ppm, the q-value from multiple t test with 5% FDR approach, the difference (fold change between disease and control), and the loading (t1), and ortholoading (to1), from OPLS-DA.

| **Metabolite** | **^1^H chemical shift (ppm)** | **Q value** | **Difference (Disease-Control)** | **Loading (t1)** | **OrthoLoading (to1)** |
| --- | --- | --- | --- | --- | --- |
| Glucose + Glycerol | 3.566 | 0.010 | 0.010 | 1.944 | 0.138 |
| Valine | 0.985 | 0.090 | -0.007 | -2.922 | -0.419 |
| Lactate | 1.306 | >0.999 | 0.004 | 1.357 | 0.099 |
| Valine | 0.961 | >0.999 | -0.003 | -1.815 | -0.340 |
| Creatine | 3.000 | >0.999 | -0.003 | -1.926 | -0.492 |
| Glutamine | 2.456 | >0.999 | -0.002 | -1.367 | -0.306 |
| Urea | 5.741 | >0.999 | 0.001 | 1.469 | 0.434 |
| Acetate | 1.904 | >0.999 | -0.001 | -0.953 | -0.297 |
| Acetamide | 2.062 | >0.999 | -0.001 | -0.344 | -0.050 |
| Alanine | 1.475 | >0.999 | -0.001 | -0.496 | -0.120 |
| 4-Aminobenzoic_acid | 7.041 | >0.999 | 0.000 | 0.067 | 0.046 |

**Supplementary table 4. Result of univariate and multivariate analysis for each bucket.** From left to right column, metabolite name or if it is unknown, it´s written the ^1^H chemical shift value in ppm. P value, difference, t ratio, df, and q value, were generated by multiple t test, with 5% FRD approach, assuming same SD between classes and peaks intensities. Loading (t1) and ortholoading (to1), were generated by OPLS-DA.

| **Metabolite** | **^1^H chemical shift (ppm)** | **P value** | **Difference (Disease-Control)** | **t ratio** | **df** | **q value** | **Loading (t1)** | **Ortho**  **Loading**  **(to1)** |
| --- | --- | --- | --- | --- | --- | --- | --- | --- |
| 0.821 | 0.821 | 0.800 | -0.00065 | 0.25 | 4212 | >0.999 | -1.07 | -0.63 |
| 0.867 | 0.867 | 0.971 | -0.00009 | 0.04 | 4212 | >0.999 | -0.20 | -0.13 |
| 0.905 | 0.905 | 0.831 | -0.00055 | 0.21 | 4212 | >0.999 | -0.70 | -0.29 |
| Valine | 0.961 | 0.217 | -0.00318 | 1.23 | 4212 | >0.999 | -1.82 | -0.34 |
| Valine | 0.985 | 0.004 | -0.00736 | 2.86 | 4212 | 0.089 | -2.92 | -0.42 |
| Valine | 1.010 | 0.481 | -0.00182 | 0.71 | 4212 | >0.999 | -1.41 | -0.37 |
| Valine | 1.036 | 0.684 | -0.00105 | 0.41 | 4212 | >0.999 | -0.92 | -0.27 |
| 1.067 | 1.067 | 0.983 | -0.00006 | 0.02 | 4212 | >0.999 | -0.12 | -0.08 |
| 1.237 | 1.237 | 0.764 | -0.00077 | 0.30 | 4212 | >0.999 | -0.95 | -0.38 |
| 1.258 | 1.258 | 0.851 | -0.00052 | 0.19 | 4212 | >0.999 | -0.86 | -0.39 |
| Lactate | 1.306 | 0.109 | 0.00413 | 1.60 | 4212 | >0.999 | 1.36 | 0.10 |
| Lactate | 1.323 | 0.170 | 0.00353 | 1.37 | 4212 | >0.999 | 1.24 | 0.09 |
| 1.356 | 1.356 | 0.599 | -0.00242 | 0.53 | 4212 | >0.999 | -1.24 | -0.35 |
| 1.390 | 1.390 | 0.947 | -0.00017 | 0.07 | 4212 | >0.999 | -0.17 | -0.09 |
| 1.428 | 1.428 | 0.997 | 0.00001 | 0.00 | 4212 | >0.999 | 0.10 | 0.07 |
| Alanine | 1.456 | 0.821 | -0.00058 | 0.23 | 4212 | >0.999 | -0.47 | -0.12 |
| Alanine | 1.475 | 0.813 | -0.00061 | 0.24 | 4212 | >0.999 | -0.50 | -0.12 |
| 1.503 | 1.503 | 0.942 | 0.00019 | 0.07 | 4212 | >0.999 | 0.31 | 0.16 |
| 1.535 | 1.535 | 0.979 | -0.00007 | 0.03 | 4212 | >0.999 | -0.17 | -0.12 |
| 1.555 | 1.555 | 0.913 | 0.00038 | 0.11 | 4212 | >0.999 | 1.00 | 0.50 |
| 1.818 | 1.818 | 0.995 | -0.00002 | 0.01 | 4212 | >0.999 | -0.07 | -0.06 |
| 1.868 | 1.868 | 0.922 | -0.00025 | 0.10 | 4212 | >0.999 | -0.23 | -0.08 |
| Acetate | 1.904 | 0.698 | -0.00100 | 0.39 | 4212 | >0.999 | -0.95 | -0.30 |
| 1.968 | 1.968 | 0.949 | 0.00016 | 0.06 | 4212 | >0.999 | 0.26 | 0.15 |
| 1.987 | 1.987 | 0.938 | 0.00020 | 0.08 | 4212 | >0.999 | 0.24 | 0.11 |
| 2.027 | 2.027 | 0.950 | 0.00016 | 0.06 | 4212 | >0.999 | 0.16 | 0.06 |
| Acetamide | 2.062 | 0.795 | -0.00067 | 0.26 | 4212 | >0.999 | -0.34 | -0.05 |
| Glutamine | 2.105 | 0.970 | 0.00010 | 0.04 | 4212 | >0.999 | -0.05 | -0.01 |
| Glutamine | 2.132 | 0.692 | -0.00102 | 0.40 | 4212 | >0.999 | -0.83 | -0.21 |
| 2.191 | 2.191 | 0.864 | 0.00055 | 0.17 | 4212 | >0.999 | 0.39 | 0.13 |
| 2.219 | 2.219 | 0.891 | -0.00035 | 0.14 | 4212 | >0.999 | -0.40 | -0.09 |
| 2.244 | 2.244 | 0.866 | -0.00043 | 0.17 | 4212 | >0.999 | -0.52 | -0.19 |
| 2.284 | 2.284 | 0.698 | -0.00100 | 0.39 | 4212 | >0.999 | -0.72 | -0.16 |
| 2.325 | 2.325 | 0.511 | -0.00169 | 0.66 | 4212 | >0.999 | -1.35 | -0.37 |
| 2.359 | 2.359 | 0.749 | -0.00082 | 0.32 | 4212 | >0.999 | -0.79 | -0.26 |
| 2.389 | 2.389 | 0.992 | 0.00002 | 0.01 | 4212 | >0.999 | 0.08 | 0.03 |
| Glutamine | 2.422 | 0.718 | 0.00093 | 0.36 | 4212 | >0.999 | 0.55 | 0.11 |
| Glutamine | 2.456 | 0.414 | -0.00210 | 0.82 | 4212 | >0.999 | -1.37 | -0.31 |
| 2.479 | 2.479 | 0.820 | -0.00059 | 0.23 | 4212 | >0.999 | -0.72 | -0.32 |
| 2.505 | 2.505 | 0.978 | -0.00007 | 0.03 | 4212 | >0.999 | -0.03 | -0.01 |
| 2.542 | 2.542 | 0.990 | 0.00003 | 0.01 | 4212 | >0.999 | 0.11 | 0.04 |
| 2.605 | 2.605 | 0.976 | -0.00008 | 0.03 | 4212 | >0.999 | -0.13 | -0.07 |
| 2.639 | 2.639 | 0.772 | -0.00074 | 0.29 | 4212 | >0.999 | -0.53 | -0.12 |
| 2.679 | 2.679 | 0.984 | 0.00005 | 0.02 | 4212 | >0.999 | 0.12 | 0.07 |
| 2.711 | 2.711 | 0.595 | -0.00137 | 0.53 | 4212 | >0.999 | -1.23 | -0.40 |
| 2.727 | 2.727 | 0.703 | -0.00099 | 0.38 | 4212 | >0.999 | -1.15 | -0.40 |
| 2.763 | 2.763 | 0.998 | 0.00001 | 0.00 | 4212 | >0.999 | 0.13 | 0.12 |
| 2.795 | 2.795 | 0.973 | 0.00009 | 0.03 | 4212 | >0.999 | 0.23 | 0.16 |
| 2.825 | 2.825 | 0.987 | 0.00004 | 0.02 | 4212 | >0.999 | 0.08 | 0.07 |
| 2.958 | 2.958 | 0.591 | -0.00141 | 0.54 | 4212 | >0.999 | -1.25 | -0.39 |
| Creatine | 3.000 | 0.312 | -0.00260 | 1.01 | 4212 | >0.999 | -1.93 | -0.49 |
| 3.026 | 3.026 | 0.248 | -0.00298 | 1.16 | 4212 | >0.999 | -2.05 | -0.44 |
| 3.064 | 3.064 | 0.912 | -0.00028 | 0.11 | 4212 | >0.999 | -0.44 | -0.20 |
| 3.112 | 3.112 | 0.633 | -0.00123 | 0.48 | 4212 | >0.999 | -0.86 | -0.20 |
| 3.160 | 3.160 | 0.951 | -0.00016 | 0.06 | 4212 | >0.999 | -0.28 | -0.17 |
| 3.184 | 3.184 | 0.923 | 0.00025 | 0.10 | 4212 | >0.999 | 0.20 | 0.07 |
| 3.211 | 3.211 | 0.513 | -0.00169 | 0.65 | 4212 | >0.999 | -1.11 | -0.22 |
| 3.234 | 3.234 | 0.177 | -0.00348 | 1.35 | 4212 | >0.999 | -1.82 | -0.30 |
| 3.254 | 3.254 | 0.153 | -0.00368 | 1.43 | 4212 | >0.999 | -1.67 | -0.23 |
| 3.290 | 3.290 | 0.820 | 0.00059 | 0.23 | 4212 | >0.999 | 0.57 | 0.20 |
| 3.339 | 3.339 | 0.793 | 0.00068 | 0.26 | 4212 | >0.999 | 0.57 | 0.12 |
| 3.369 | 3.369 | 0.429 | -0.00204 | 0.79 | 4212 | >0.999 | -1.28 | -0.26 |
| Glucose | 3.397 | 0.139 | -0.00382 | 1.48 | 4212 | >0.999 | -1.71 | -0.25 |
| Glucose | 3.431 | 0.025 | -0.00577 | 2.24 | 4212 | 0.395 | -2.10 | -0.24 |
| Glucose | 3.451 | 0.003 | -0.00772 | 3.00 | 4212 | 0.063 | -2.68 | -0.29 |
| 3.481 | 3.481 | 0.560 | -0.00150 | 0.58 | 4212 | >0.999 | -0.86 | -0.15 |
| Glucose | 3.504 | 0.367 | -0.00233 | 0.90 | 4212 | >0.999 | -1.18 | -0.19 |
| Glucose+Glycerol | 3.532 | 0.000 | 0.00927 | 3.60 | 4212 | 0.013 | 2.56 | 0.27 |
| Glycerol | 3.566 | 0.000 | 0.01017 | 3.95 | 4212 | 0.01 | 1.94 | 0.14 |
| Glycerol | 3.593 | 0.645 | 0.00119 | 0.46 | 4212 | >0.999 | 1.03 | 0.26 |
| Glycerol | 3.625 | 0.001 | 0.00836 | 3.25 | 4212 | 0.039 | 2.38 | 0.24 |
| Glycerol | 3.665 | 0.000 | 0.00948 | 3.68 | 4212 | 0.013 | 2.27 | 0.20 |
| Glucose | 3.706 | 0.951 | 0.00016 | 0.06 | 4212 | >0.999 | 0.22 | 0.02 |
| Glucose | 3.760 | 0.007 | 0.00693 | 2.69 | 4212 | 0.128 | 2.17 | 0.25 |
| Glucose | 3.787 | 0.032 | 0.00554 | 2.15 | 4212 | 0.441 | 1.89 | 0.23 |
| Glucose | 3.821 | 0.117 | 0.00404 | 1.57 | 4212 | >0.999 | 1.85 | 0.27 |
| Glucose | 3.848 | 0.658 | 0.00114 | 0.44 | 4212 | >0.999 | 0.57 | 0.10 |
| Glucose | 3.902 | 0.646 | 0.00118 | 0.46 | 4212 | >0.999 | 0.52 | 0.08 |
| 3.936 | 3.936 | 0.911 | 0.00029 | 0.11 | 4212 | >0.999 | 0.33 | 0.12 |
| 3.977 | 3.977 | 0.755 | -0.00080 | 0.31 | 4212 | >0.999 | -0.82 | -0.30 |
| 4.009 | 4.009 | 0.672 | -0.00154 | 0.42 | 4212 | >0.999 | -0.66 | -0.23 |
| 4.042 | 4.042 | 0.863 | 0.00045 | 0.17 | 4212 | >0.999 | 0.71 | 0.32 |
| Lactate | 4.073 | 0.790 | 0.00069 | 0.27 | 4212 | >0.999 | 0.88 | 0.33 |
| Lactate | 4.099 | 0.163 | 0.00359 | 1.40 | 4212 | >0.999 | 1.93 | 0.30 |
| Lactate | 4.126 | 0.786 | 0.00070 | 0.27 | 4212 | >0.999 | 0.88 | 0.32 |
| 4.218 | 4.218 | 0.999 | 0.00001 | 0.00 | 4212 | >0.999 | 0.17 | 0.22 |
| 4.238 | 4.238 | 0.999 | 0.00000 | 0.00 | 4212 | >0.999 | 0.19 | 0.21 |
| 5.088 | 5.088 | 0.993 | -0.00003 | 0.01 | 4212 | >0.999 | -0.10 | -0.14 |
| 5.173 | 5.173 | 1.000 | 0.00000 | 0.00 | 4212 | >0.999 | 0.07 | 0.10 |
| Glucose | 5.218 | 0.776 | 0.00073 | 0.28 | 4212 | >0.999 | 0.68 | 0.21 |
| Glucose | 5.228 | 0.770 | 0.00075 | 0.29 | 4212 | >0.999 | 0.71 | 0.22 |
| 5.320 | 5.320 | 0.994 | -0.00004 | 0.01 | 4212 | >0.999 | -0.38 | -0.59 |
| 5.429 | 5.429 | 0.994 | 0.00002 | 0.01 | 4212 | >0.999 | 0.05 | 0.05 |
| 5.523 | 5.523 | 0.996 | -0.00001 | 0.01 | 4212 | >0.999 | -0.13 | -0.06 |
| 5.657 | 5.657 | 0.893 | -0.00043 | 0.13 | 4212 | >0.999 | -1.24 | -0.30 |
| Urea | 5.741 | 0.564 | 0.00149 | 0.58 | 4212 | >0.999 | 1.47 | 0.43 |
| 6.325 | 6.325 | 0.996 | -0.00002 | 0.00 | 4212 | >0.999 | 0.00 | -0.01 |
| 6.827 | 6.827 | 0.998 | -0.00001 | 0.00 | 4212 | >0.999 | -0.02 | -0.02 |
| 6.876 | 6.876 | 0.977 | -0.00007 | 0.03 | 4212 | >0.999 | -0.18 | -0.15 |
| 6.897 | 6.897 | 0.963 | -0.00012 | 0.05 | 4212 | >0.999 | -0.26 | -0.19 |
| 6.966 | 6.966 | 0.982 | 0.00007 | 0.02 | 4212 | >0.999 | 0.36 | 0.45 |
| 4-Aminobenzoic_acid | 7.041 | 0.949 | -0.00019 | 0.06 | 4212 | >0.999 | 0.07 | 0.05 |
| 7.172 | 7.172 | 0.967 | -0.00011 | 0.04 | 4212 | >0.999 | -0.24 | -0.18 |
| 7.193 | 7.193 | 0.978 | -0.00007 | 0.03 | 4212 | >0.999 | -0.17 | -0.15 |
| 7.257 | 7.257 | 0.997 | 0.00001 | 0.00 | 4212 | >0.999 | 0.05 | 0.07 |
| 7.276 | 7.276 | 0.992 | 0.00003 | 0.01 | 4212 | >0.999 | 0.15 | 0.25 |
| 7.312 | 7.312 | 0.983 | -0.00006 | 0.02 | 4212 | >0.999 | -0.12 | -0.10 |
| 7.365 | 7.365 | 0.964 | -0.00012 | 0.05 | 4212 | >0.999 | -0.26 | -0.17 |
| 7.382 | 7.382 | 0.978 | -0.00007 | 0.03 | 4212 | >0.999 | -0.13 | -0.07 |
| 7.421 | 7.421 | 0.979 | -0.00007 | 0.03 | 4212 | >0.999 | -0.13 | -0.09 |
| 7.524 | 7.524 | 0.982 | -0.00006 | 0.02 | 4212 | >0.999 | -0.19 | -0.23 |
| 7.544 | 7.544 | 0.916 | -0.00027 | 0.11 | 4212 | >0.999 | -0.41 | -0.20 |
| 7.601 | 7.601 | 0.865 | -0.00054 | 0.17 | 4212 | >0.999 | -0.33 | -0.16 |
| 7.618 | 7.618 | 0.974 | 0.00014 | 0.03 | 4212 | >0.999 | 0.04 | 0.02 |
| 7.657 | 7.657 | 0.992 | -0.00003 | 0.01 | 4212 | >0.999 | -0.12 | -0.18 |
| 7.715 | 7.715 | 0.991 | -0.00003 | 0.01 | 4212 | >0.999 | -0.04 | -0.06 |
| 7.738 | 7.738 | 0.918 | 0.00026 | 0.10 | 4212 | >0.999 | 0.46 | 0.27 |
| 7.765 | 7.765 | 0.931 | -0.00032 | 0.09 | 4212 | >0.999 | -0.41 | -0.24 |
| 7.831 | 7.831 | 0.976 | -0.00008 | 0.03 | 4212 | >0.999 | -0.16 | -0.18 |
| 7.852 | 7.852 | 0.659 | -0.00139 | 0.44 | 4212 | >0.999 | -0.76 | -0.27 |
| 7.923 | 7.923 | 0.977 | -0.00007 | 0.03 | 4212 | >0.999 | -0.33 | -0.40 |
| 8.182 | 8.182 | 0.990 | 0.00005 | 0.01 | 4212 | >0.999 | 0.22 | 0.24 |
| 8.443 | 8.443 | 0.998 | -0.00001 | 0.00 | 4212 | >0.999 | 0.04 | 0.03 |
| 9.267 | 9.267 | 0.944 | -0.00018 | 0.07 | 4212 | >0.999 | -0.48 | -0.35 |
